# Role of the Metal Cation on the Amplified Spontaneous Emission Properties of Two-Dimensional Perovskites

**DOI:** 10.1021/jacs.5c13296

**Published:** 2025-11-20

**Authors:** Yarong He, E Laine Wong, Nurgul Sarsembek, Ranita Samanta, Davide Regaldo, Andrea Pianetti, Michela Cecconi, Guglielmo Lanzani, Annamaria Petrozza, Daniele Cortecchia

**Affiliations:** † Center for Nano Science and Technology @Polimi, Istituto Italiano di Tecnologia, via Rubattino 81, Milano 20134, Italy; ‡ Dipartimento di Fisica, Politecnico di Milano, Milano 20133, Italy; § Department of Industrial Chemistry “Toso Montanari”, University of Bologna, via Piero Gobetti 85, 40129 Bologna, Italy

## Abstract

Two-dimensional (2D) perovskites are attracting renewed interest for coherent emission. While excellent results have been achieved with tin-based materials, the real applicability of lead perovskites remains controversial due to limited reproducibility. Critically, there is no fundamental explanation so far to provide an educated prediction about their lasing properties. In this work, we compare the 2D perovskites PEA_2_PbI_4_, PEA_2_SnI_4_, and their mixed compositions. Photophysical characterization and solid-state nuclear magnetic resonance (ssNMR) spectroscopy reveal that molecular motions of the organic cation and local lattice disorder induced by metal cation mixing critically impact the amplified spontaneous emission (ASE) properties of the material. We show that ASE can be achieved for both perovskites at 78 K employing short pump pulse width (fs) and near-band gap excitation. However, stable operation at room temperature is achieved only in PEA_2_SnI_4_, thanks to the lower Auger recombination rate, its peculiar lengthening of the excited state lifetime at higher temperatures and lower impact of trap-mediated recombination compared to PEA_2_PbI_4_. Our work highlights the importance of defect control and crystal engineering strategies to enhance the structural rigidity and improve the optoelectronic properties of this class of soft semiconductors.

## Graphical abstract

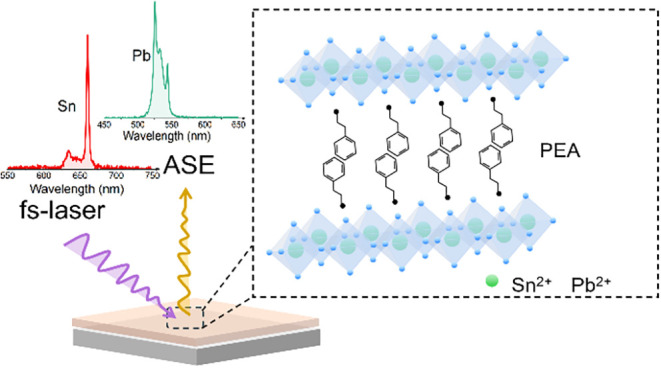

## Introduction

Metal halide perovskites are promising semiconductors for photonic sources, owing to their wide spectral tunability, high efficiency, and facile solution-processability. These features make them particularly attractive for the integration with existing silicon photonic platforms, also offering viable alternatives to fill the “green gap” in semiconductor lasers.^1,2^ Three-dimensional (3D) perovskites have made great strides since the first pioneering demonstration of amplified spontaneous emission (ASE) and lasing in 2014,^3,4^ reaching the realization of perovskite lasers under continuous-wave (CW) optical pumping conditions.^5,6^ Meanwhile, 2D perovskites are being explored to improve the material’s stability, and their high exciton binding energy (EBE) has often been considered helpful to boost radiative emission.^7–9^ Historically, PEA_2_PbI_4_ (PEA = phenylethylammonium) was the first of this class of materials to be proposed for lasing applications, where coherent emission was claimed at 16 K as early as 1998.^10^ Since then, a few reports have claimed lasing in other 2D perovskites, including OA_2_PbI_4_ (OA = octylamine),^11^ BA_2_PbI_4_ (BA = butylammonium),^12^ and PEA_2_PbI_4_ (PEA = phenylethylammonium).^13^ However, considering the Ruddlesden–Popper series A′_2_A_*n*–1_Pb*_n_* X_3*n*+1_,^14^ several other works reported the impossibility of reaching lasing in the *n* = 1 layered lead perovskites, highlighting an increase in lasing threshold with the reduction in dimensionality from high toward small *n* values, leading to the conclusion that the material would undergo degradation before reaching the conditions for population inversion.^15–18^ To the best of our knowledge, ASE studies, a useful tool for characterization of gain media, on 2D lead perovskite films have not been reported to date. In 2022, we demonstrated that 2D tin perovskites are good gain media through the realization of a DFB laser based on PEA_2_SnI_4_ thin films working at 80 K; stable room temperature operation in 2D tin perovskite thin films remained a challenge.^19^ Subsequent work showed lasing in 2D tin perovskite microcrystals (298 K) and nanowires (88 K),^20–22^ further confirming the excellent optical gain properties of tin-based compounds. However, this leaves unsolved the question of the fundamental properties underlying the markedly different behavior of lead and tin compounds. Understanding these differences is a key aspect, since difficulties in reproducibility have frustrated the advancement of the field for more than 20 years.

In this work, we compare PEA_2_PbI_4_ and PEA_2_SnI_4_ and further assess the evolution of their properties in the mixed compositions PEA_2_Sn_*x*_Pb_1–*x*_I_4_. We investigate the ASE properties in thin films via power- and temperature-dependent photoluminescence measurements under various optical pumping conditions. We further combine time-resolved photoluminescence and solid-state nuclear magnetic resonance (ssNMR) spectroscopy to investigate the exciton recombination dynamics as well as the ^13^C spin–lattice recombination dynamics of the organic component. Structurally, the aromatic ring of the phenylethylammonium imparts increased structural rigidity to the perovskites, allowing both compounds to reach population inversion and sustain ASE at 78 K under ideal pumping conditions. In both perovskites, we observe a fast exponential growth of the ASE threshold with temperature, likely connected to the fast molecular motion of the ethylammonium moiety, causing rapid ASE thermal quenching. However, while we find that the performance of the Pb-based compound is worsened by a series of factors leading to ASE quenching above 120 K, such as an increased Auger recombination, shortening of the excited state lifetime, and an increase in trapping rates at defects, we show that stable ASE at room temperature can be achieved in PEA_2_SnI_4_ thin films. This behavior is attributed to the peculiar lengthening of photoluminescence lifetime with increasing temperature, endorsing 2D tin perovskites as excellent candidates for photonic applications. Future developments should target defect control strategies and molecular engineering to increase the structural rigidity to further enhance the properties of this class of low-dimensional semiconductors.

## Results and Discussion

Figure 1a schematically illustrates the structure of layered perovskites PEA_2_XI_4_ (where PEA = phenylethylammonium and X = Pb^2+^, Sn^2+^), which are composed of a multilayer architecture of alternating organic and inorganic sheets. The inorganic network consists of layers of corner-sharing [Pb/SnI_6_]^4–^ octahedra, separated by PEA cations that serve as organic spacers. Powder X-ray diffraction confirmed the formation of the desired phases, consistent with the previously reported triclinic crystal structures,^23,24^ which yield very similar diffraction patterns for both the lead and tin perovskite (Figure S1). For a deeper investigation of the structural properties and local coordination environment of the materials, we performed solid-state NMR measurements.

**Figure 1:**
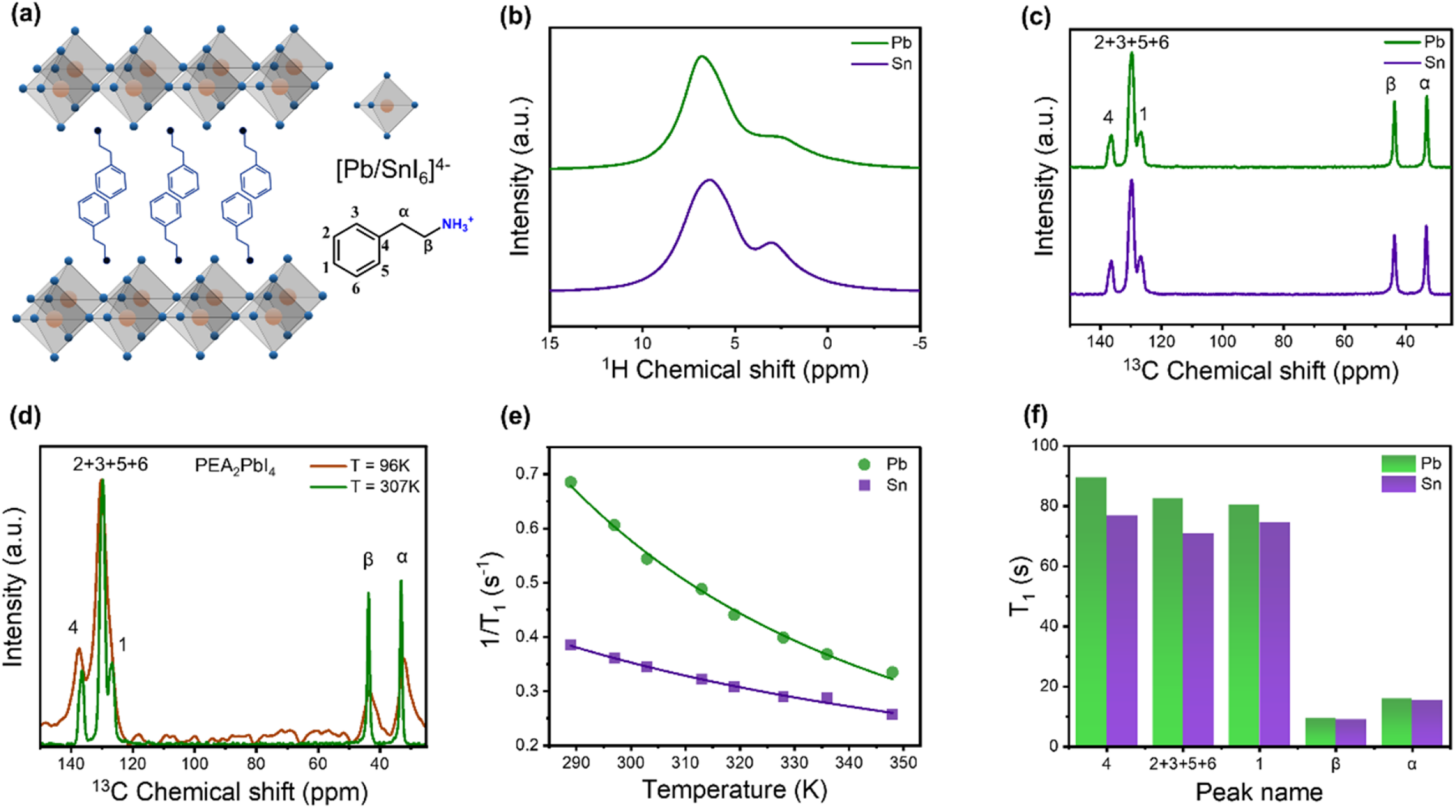
Solid-state NMR characterization of the organic part of PEA_2_XI_4_ (X = Pb, Sn). (a) Schematic illustration of the 2D layered perovskite structure, consisting of alternating organic (PEA) and inorganic ([Pb/SnI_6_]^4–^) layers. MAS ssNMR spectra for PEA_2_PbI_4_ (green), PEA_2_SnI_4_ (purple): (b) ^1^H spectra at 42 kHz MAS rate (c) ^13^C CP-MAS spectra at 12 kHz MAS rate. (d) Comparison of ^13^C spectra of PEA_2_PbI_4_ at 96 K (brown) and 307 K (green). (e) Temperature-dependence of ^1^H spin–lattice relaxation times (1/T_1_) for PEA_2_PbI_4_ (green) and PEA_2_SnI_4_ (purple). (f) ^13^C spin–lattice relaxation times (T_1_) measured for PEA_2_PbI_4_ (green) and PEA_2_SnI_4_ (purple) at room temperature.

In the ^1^H spectrum measured at 42 kHz magic angle spinning (MAS) frequency, two bands can be distinguished that can be attributed to the aliphatic and aromatic part of PEA centered at 2.9 and 6.5 ppm, respectively (Figure 1b). ^13^C spectra were measured via cross-polarization at 12 kHz MAS rate and allowed to better distinguish signals corresponding to the aromatic ring between 140 and 120 ppm, and the more shielded signals of the ethyl chain between 50 and 25 ppm (Figure 1c). The high similarity between the spectra of PEA_2_PbI_4_ and PEA_2_SnI_4_ for both ^1^H and ^13^C indicates no major structural differences between the lead and tin perovskite in the packing of the organic layer. In the case of PEA_2_PbI_4_, we additionally probed the ^13^C spectrum at lower temperature to verify any structural difference occurring at temperatures which are relevant for its working regime for amplified spontaneous emission (occurring below 120 K, see later sections). At 96 K, there is no evident shift in the ^13^C signals upon cooling (Figure 1d). However, we observe a two and six-times increase in full width at half-maximum (fwhm) of the ^13^C peaks of the aromatic and aliphatic moieties, respectively, from 307 to 96 K. This occurs as a direct consequence of a shorter transverse relaxation time (*T*_2_) at lower temperature (according to the relation fwhm = 1/π*T*_2_). *T*_2_ relaxation is primarily influenced by the random fluctuations of local magnetic fields caused by molecular motions. As the temperature decreases, the molecular reorientation rate slows down,^25^ leading to structural rigidification and a stronger dipolar interaction between the nuclear spins. Consequently, spin–spin relaxation is enhanced as spins lose phase coherence more rapidly due to the spin dephasing, resulting in a shorter *T*_2_.^25^ The marked broadening of ^13^C peaks at 78 K thus highlights a significant rigidification, in particular of the ethyl group.

We further assessed the molecular motions in the lead and tin perovskites via spin–lattice relaxation measurements (Figure 1e,f). Notably, the structural rigidity imparted by the organic templating cation of layered perovskites has been recognized as a critical determinant of both efficient luminescence and low lasing thresholds,^18,26^ and spin relaxation-based analysis has previously been applied to assess crystal rigidity in layered low-dimensional perovskites.^27^ The spin–lattice relaxation time *T*_1_, a probe for the fast molecular motions occurring at a frequency close to the Larmor frequency in the MHz range, reflects the efficiency of energy exchange between nuclear spins and their surrounding lattice.^28^ In the case of ^1^H, *T*_1_ tends to equilibrate due to extensive ^1^H–^1^H dipolar networks and fast spin diffusion, and therefore it is generally not possible to discern individual relaxation behaviors for different parts of the molecule, but rather yields an average picture of the molecular motion.^28^ The ^1^H *T*_1_ of the two perovskites under investigation shows a decrease with temperature characteristic of a fast time scale motional regime^29^ (Figure 1e). The fitting of this trend with the Bloembergen-Purcell-Pound (BPP) model allows for to retrieval of the activation energy for the molecular motion, yielding 11.1 ± 1.8 kJ/mol and 6.1 ± 1.9 kJ/mol for PEA_2_PbI_4_ and PEA_2_SnI_4_, respectively (see description in methods section SI and Table S1). The higher energy needed for the thermal activation of the molecular motions thus suggests a more rigid structural environment in the lead than in the tin perovskite. We further investigated the spin relaxation dynamics of ^13^C nuclei. Since the spin relaxation of ^13^C is closely related to the dipolar coupling to ^1^H and the locally oscillating magnetic field, and the lower isotopic abundance mitigates the effect of spin diffusion and homonuclear dipolar coupling compared to ^1^H, it can be used to probe the molecular mobility at each specific position of the organic molecule.^30,31^ Considering that *T*_1_ is affected by both local proton density and molecular dynamics, in this context, its interpretation can provide a qualitative assessment of the molecular motion. At room temperature, the aromatic ring exhibits remarkably longer *T*_1_ of 60–90 s, while the ethyl group relaxes more efficiently in the range of 10–20 s, albeit longer than for simpler alkylammonium chains.^28,30^ The same relative trend is maintained through the investigated temperature range (260–330 K, see Figure S2), with an overall slow decrease of *T*_1_ values with increasing temperature. The much shorter *T*_1_ of the ethylammonium linker may be interpreted as a higher mobility of this moiety, consistent with the localized torsional motion arising from the rotation of the C_α_–C_β_ and C_4_–C_α_ bonds identified through ^1^H NMR in previous works.^32^ On the other hand, the results highlight the effectiveness of the aromatic ring to provide a more rigid structural framework. Comparing the two perovskites, despite an overall similarity, we observe slightly longer relaxation times in PEA_2_PbI_4_, possibly connected to a higher structural rigidity, as also inferred from the analysis of ^1^H relaxation times.

To gain a deeper insight into the inorganic framework, we additionally probed the ^207^Pb and ^119^Sn nuclei of PEA_2_PbI_4_, PEA_2_SnI_4_, and the mixed Pb/Sn composition PEA_2_Pb_0.5_Sn_0.5_I_4_ via static ssNMR (Figure 2a,b). From the line shape analysis of their spectral response, we extracted the corresponding NMR tensor parameters, namely the isotropic chemical shift (δ_iso_), chemical shift anisotropy (Δδ), and asymmetry index (η) (Table 1).

**Figure 2:**
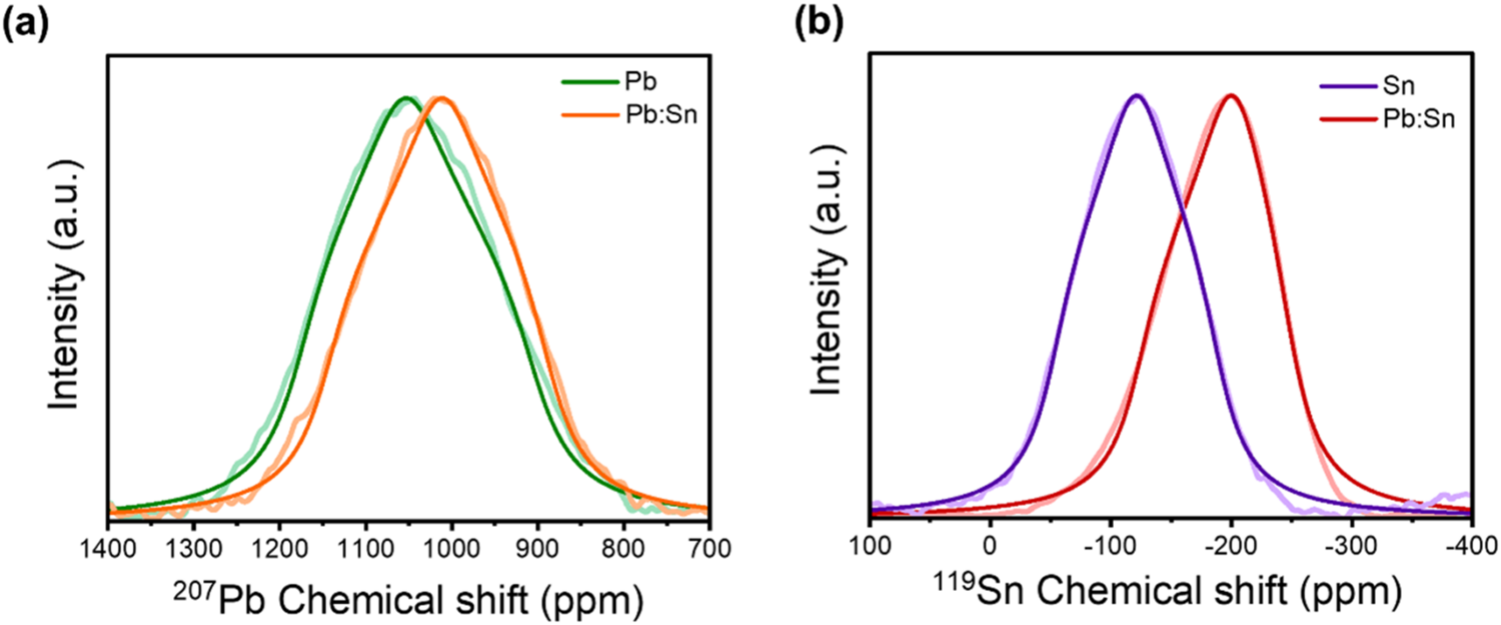
Solid-state NMR characterization of the inorganic part of the perovskites. (a) ^207^Pb static ssNMR spectra and corresponding fits for PEA_2_PbI_4_ (green), PEA_2_Pb_0.5_Sn_0.5_I_4_ (orange). (b) ^119^Sn static ssNMR spectra and fits for PEA_2_SnI_4_ (purple), PEA_2_Pb_0.5_Sn_0.5_I_4_ (red).

**Table 1:** ^207^Pb and ^119^Sn Isotropic Chemical Shifts (δ_iso_), Chemical Shift Anisotropy (Δδ), Asymmetry Parameter (η) Defined According to the Haeberlen Convention of PEA_2_XI_4_ (X = Pb, Sn) Perovskites

sample	^207^Pb, δ_iso_ (ppm)	^119^Sn, δ_iso_ (ppm)	Δδ (ppm)	η
PEA_2_PbI_4_	1045.97	-	–138.83	0.872
PEA_2_Pb_0.5_Sn_0.5_I_4_	1014.90	-	128.49	0.943
	-	–120.65	67.06	0.984
PEA_2_SnI_4_	-	–190.17	66.57	0.677

The partial replacement of lead with tin shifts the ^207^Pb resonance from 1046 to 1015 ppm and reduces its CSA from −138 to 128 ppm. Concerning ^119^Sn, a considerable shift is observed from PEA_2_Pb_0.5_Sn_0.5_I_4_ (−120 ppm) to PEA_2_SnI_4_ (−190 ppm), while a smaller CSA of ∼ 67 ppm is maintained for both compounds. This decreasing trend in both isotropic chemical shift and anisotropy with the increase of tin content can be rationalized by the intrinsic electronic differences between Pb^2+^ and Sn^2+^. The lighter Sn cation exhibits weaker relativistic spin–orbit coupling,^33^ which can diminish the anisotropy of the shielding tensor. In addition, the shallower and more active Sn-5s lone-pair states promote stronger antibonding s–p coupling with I-5p orbitals than the deeper Pb-6s states, producing enhanced hybridization and a more dispersive valence-band edge.^34,35^ This enhanced orbital overlap strengthens the covalent character of Sn–I bonds relative to the more ionic Pb–I framework,^35^ homogenizing the electronic density around the Sn nucleus, contributing to the reduced CSA. Consistently, the asymmetry index (η) increases in the mixed systems (η = 0.943 for ^207^Pb, 0.984 for ^119^Sn), reflecting local lattice disorder imposed by metal cation mixing. In contrast, the pure Sn framework relaxes into a more symmetric coordination environment, as evidenced by the reduced η value (0.677).

We then investigated the amplified spontaneous emission properties in the perovskite thin films. These were made via spin coating by preparing 0.2 M solutions of the precursors phenethylammonium iodide (PEA)I and lead iodide (PbI_2_)/tin iodide (SnI_2_) in *N*,*N*-dimethylformamide (DMF). X-ray diffraction analysis confirms perovskite formation, with a predominance of a single diffraction peak around 5.3°, indicating a strong preferential orientation of the perovskite toward the 002 direction, Figure S3a.^36^ The deposition was optimized to achieve similar film morphologies for both PEA_2_PbI_4_ and PEA_2_SnI_4_, which are characterized by grains of the order of 100 nm and a similarly low surface roughness of about 2–3 nm, as confirmed by SEM and AFM characterization Figure S3b,c.

Both thin films exhibit the characteristic optical features of 2D perovskites, with sharp excitonic absorption peaks located at 516 nm (PEA_2_PbI_4_) and 612 nm (PEA_2_SnI_4_), followed by distinct onsets of interband absorption (Figure 3a,b). Upon photoexcitation, narrowband emission is observed at 525 and 625 nm, corresponding to green and red photoluminescence, respectively. Notably, PEA_2_PbI_4_ also displays a weak broadband emission around 750 nm. The analysis of the PL thermal quenching in the 78–293 K temperature range allows the retrieval of the exciton–phonon coupling strengths for both PEA_2_PbI_4_ (150 ± 105 meV) and PEA_2_SnI_4_ (570 ± 292 meV) by fitting the fwhm temperature dependence (Figures S4–S8). The lower exciton–phonon coupling for the lead compound correlates well with the higher structural rigidity compared to PEA_2_SnI_4_, as evinced from the ssNMR measurements previously discussed.

**Figure 3:**
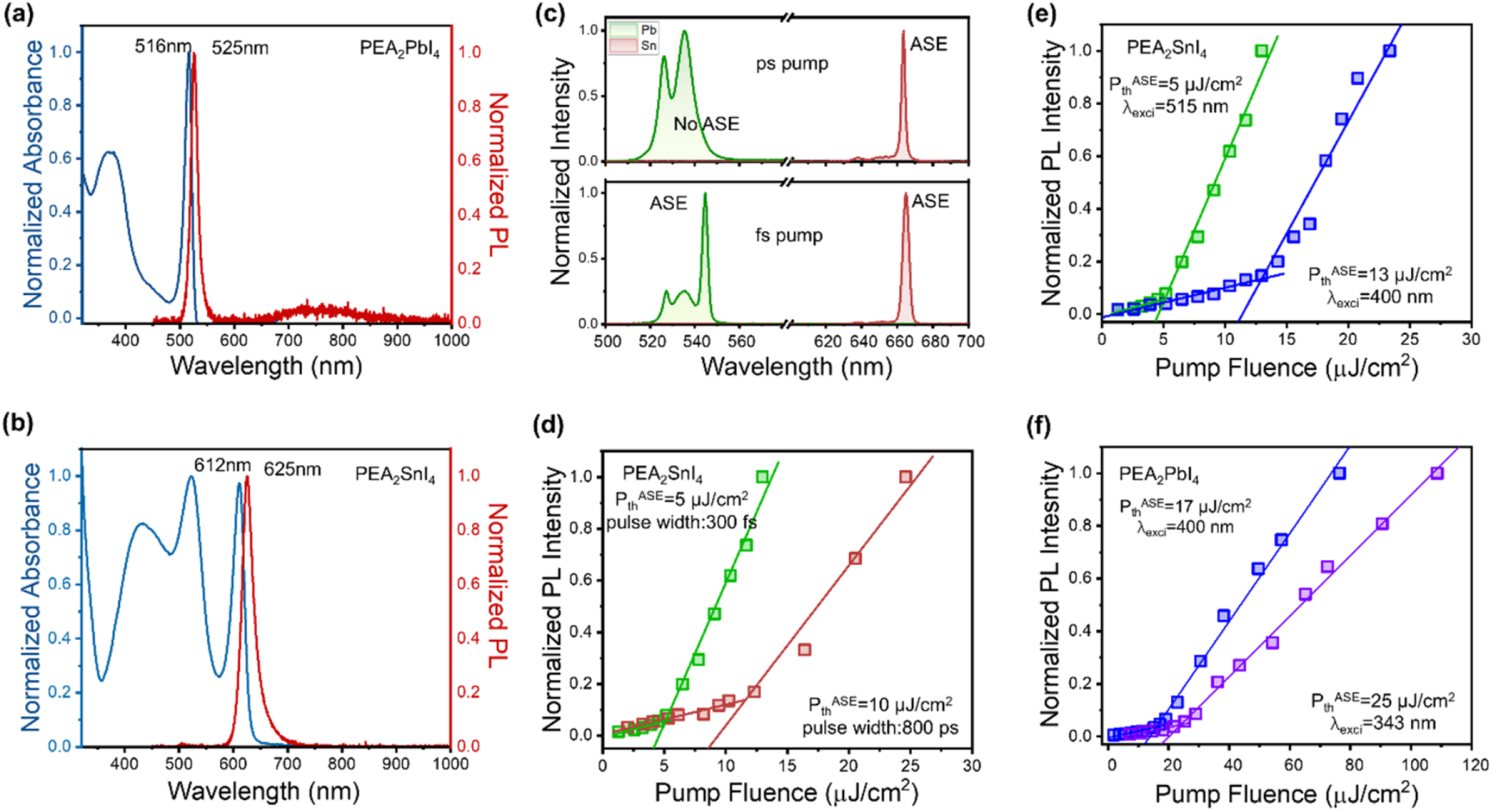
Optical Characterization and Amplified Spontaneous Emission in PEA_2_SnI_4_ and PEA_2_PbI_4_ at 78 K. (a) The UV–vis absorption and photoluminescence spectra of PEA_2_PbI_4_ and (b) PEA_2_SnI_4_ thin films at room temperature. (c) Emission spectra under ps (upper panel) optical pump (fluence 25 μJ/cm^2^, excitation wavelength 532 nm for Sn; fluence 589 μJ/cm^2^, excitation wavelength 355 nm for Pb) and fs (lower panel) optical pump (fluence 13 μJ/cm^2^, excitation wavelength 515 nm for Sn; fluence 109 μJ/cm^2^ excitation wavelength 343 nm for Pb). Peak intensity evolution as a function of the excitation fluence: (d) PEA_2_SnI_4_ as a function of the excitation conditions (red pulse width 800 ps, an excitation wavelength of 532 nm, green pulse width 300 fs, an excitation wavelength of 515 nm). (e) PEA_2_SnI_4_ as a function of the excitation energy (blue 400 nm, green 515 nm) with fixed excitation pulse width 300 fs. (f) PEA_2_PbI_4_ as a function of the excitation energy (blue 400 nm, purple 343 nm) with fixed excitation pulse width 300 fs.

To minimize the effect of nonradiative losses due to molecular motions, we first performed power-dependent luminescence measurements at 78 K. At low temperature, the photoluminescence of both materials shows a finer structure, which has previously been attributed to the presence of exciton polarons (Figures S4–S8).^37^ In addition to this, under optical pumping, we observed the development of amplified spontaneous emission (ASE) for both materials, peaked at 665 nm in PEA_2_SnI_4_ and 545 nm in PEA_2_PbI_4_, respectively (Figures 3c, S9 and 10). We find the ASE characteristics to be critically dependent on the optical excitation conditions. In PEA_2_SnI_4_ thin films, excitation with a pulse width of 800 ps laser results in an ASE threshold of 10 μJ/cm^2^, while this can be lowered to 5 μJ/cm^2^ by using a shorter pulse width of 300 fs (Figures 3d and S9). In PEA_2_PbI_4_, this difference is even more remarkable, since ASE could only be achieved under fs pumping (Figure S11). This can be explained considering that the higher peak power of fs pulses results in a higher instantaneous carrier density, which shall favor population inversion via a faster population of the excited state. On the contrary, the 800 ps pump will allow a longer time for the depopulation of the excited state (having a lifetime generally below 500 ps in the two perovskites, see later discussion of Figure 5a,b, and Table S2) via spontaneous radiative decay and other nonradiative processes.

By maintaining the fs pumping conditions, we further investigated the effect of the excitation wavelength by comparing excitation close to the band edge versus higher energy excitations (Figures 3e,f, S9 and 10). For PEA_2_SnI_4_, green excitation (515 nm) results in a 65% decrease of ASE threshold compared to blue excitation (400 nm), as shown in Figure 3e. Likewise, the ASE threshold undergoes a 32% decrease from 343 to 400 nm excitation in PEA_2_PbI_4_ (Figure 3f). This trend highlights a more efficient carrier injection when the excitation is near the band edge: in fact, the reduction in the excess kinetic energy minimizes the need for carriers to undergo phonon-mediated relaxation before participating in recombination processes, during which they may more likely undergo additional nonradiative processes and dissipate energy as heat rather than contributing to stimulated emission (Figure S12). This translates into a more efficient population inversion, thus favoring ASE. To push toward CW optical pumping, we were able to achieve ASE for both films with no significant increase in their thresholds up to 500 kHz at 78 K, showing the absence of additional sample heating due to the higher number of laser pulses, as long as the photocarrier lifetime is shorter than the laser pulse interval (Figures S13 and S14).

Despite the similarities, we always observe a better performance in the tin than in the lead perovskite. In the same conditions of fs pump near band edge, PEA_2_SnI_4_ has a lower threshold (5 vs 17 μJ/cm^2^) and higher ASE slope efficiency, which results in a much higher ASE intensity relative to the spontaneous emission (Figure 3). By measuring and statistically analyzing over 15 samples of both Sn- and Pb-based perovskite materials, we also found that the threshold values distribution of Pb perovskite is broader than that of Sn perovskite (Figure S15). At first, this is surprising given that one may expect an opposite trend for the ASE thresholds, given the higher structural rigidity and lower exciton–phonon coupling for PEA_2_PbI_4_ discussed above, which shall favor radiative recombination and ASE,^18,26^ and suggests a more complicated framework where other factors shall come into play.

Among these, from low-temperature optical absorption, we retrieved significantly different exciton binding energies (EBE) for the two perovskites, corresponding to 100 and 200 meV for PEA_2_SnI_4_ and PEA_2_PbI_4_, respectively (Figure S16). The enhanced electron–hole Coulomb interaction caused by the high EBE is known to correlate with rapid Auger recombination, since it causes a nonuniform spatial distribution of the carriers at high excitation densities, which accelerates the Auger recombination process, whose rate typically increases with the EBE.^38–43^ In one-dimensional semiconductors, for instance, it has been shown that the Auger recombination rate scales with the cube of the EBE,^44^ while it has been identified as a key factor limiting the performance of multidimensional perovskites and CsPbBr_3_ nanoplatelets for light-emitting diodes and lasing applications.^45,46^ Recent reports on lead-based perovskites have shown a lowering of the lasing threshold with increasing perovskite dimensionality and suggest that a stronger quantum confinement effect in the smaller-n perovskites could hamper the realization of population inversion, thus resulting in higher thresholds.^8,17^ To verify this in our systems, we performed relative photoluminescence quantum yield (PLQY) measurements (Figure S17, room temperature data), which confirm that the higher EBE causes Auger recombination to start at excitation densities 1 order of magnitude lower for PEA_2_PbI_4_ (1.81 × 10^16^ cm^–3^) than for PEA_2_SnI_4_ (1.21 × 10^17^ cm^–3^). In our measurements, the ASE regime starts at the excitation density of 1.49 × 10^18^ (PEA_2_PbI_4_) and 4.51 × 10^17^ (PEA_2_SnI_4_) at 78 K, and further increases with temperature up to 3.98 × 10^18^ cm^–3^ (PEA_2_PbI_4_, at the highest working temperature of 118 K) and 1.84 × 10^19^ cm^–3^ (PEA_2_SnI_4_, 293 K) (see later discussion on the temperature-dependent analysis). Therefore, we conclude that Auger recombination can significantly worsen the contribution of nonradiative losses to the depopulation of the excited state, particularly affecting the lead-based perovskite with the most negative impact.^17,45^

To gain a deeper insight into the effect of the metal cations, we further investigated the series of PEA_2_Sn_*x*_Pb_1–*x*_I_4_ by gradual substitution of tin with lead (Figures 4, S18 and 19). Here, the composition corresponds to the nominal stoichiometry, considering the starting molar ratio of the precursors used in the preparation. As shown in Figures S20 and S21, the morphology is only slightly affected by the substitution: the surface roughness remains around 2–3 nm, although an increase in porosity with the lead content is shown by the SEM and AFM analysis (Figures S20 and S21). From the optical absorption at room temperature, we observed that as tin is gradually replaced by lead (*x* = 1.0–0.4), the exciton peak shifts from 612 to 583 nm, accompanied by a reduction in its intensity (Figure 4a); this change in optical properties is expected based on the alloying properties observed with ssNMR, that confirm the atomic-scale intermixing of the two metal cations in the perovskite framework. In the range *x* = 0.3–0.0, we observed phase disproportionation, as evidenced by the coexistence of the excitonic peaks of both the tin-rich phase (573 nm) and the lead-rich phase (516 nm), suggesting an incompatibility in the crystallization kinetics at high lead contents.

**Figure 4:**
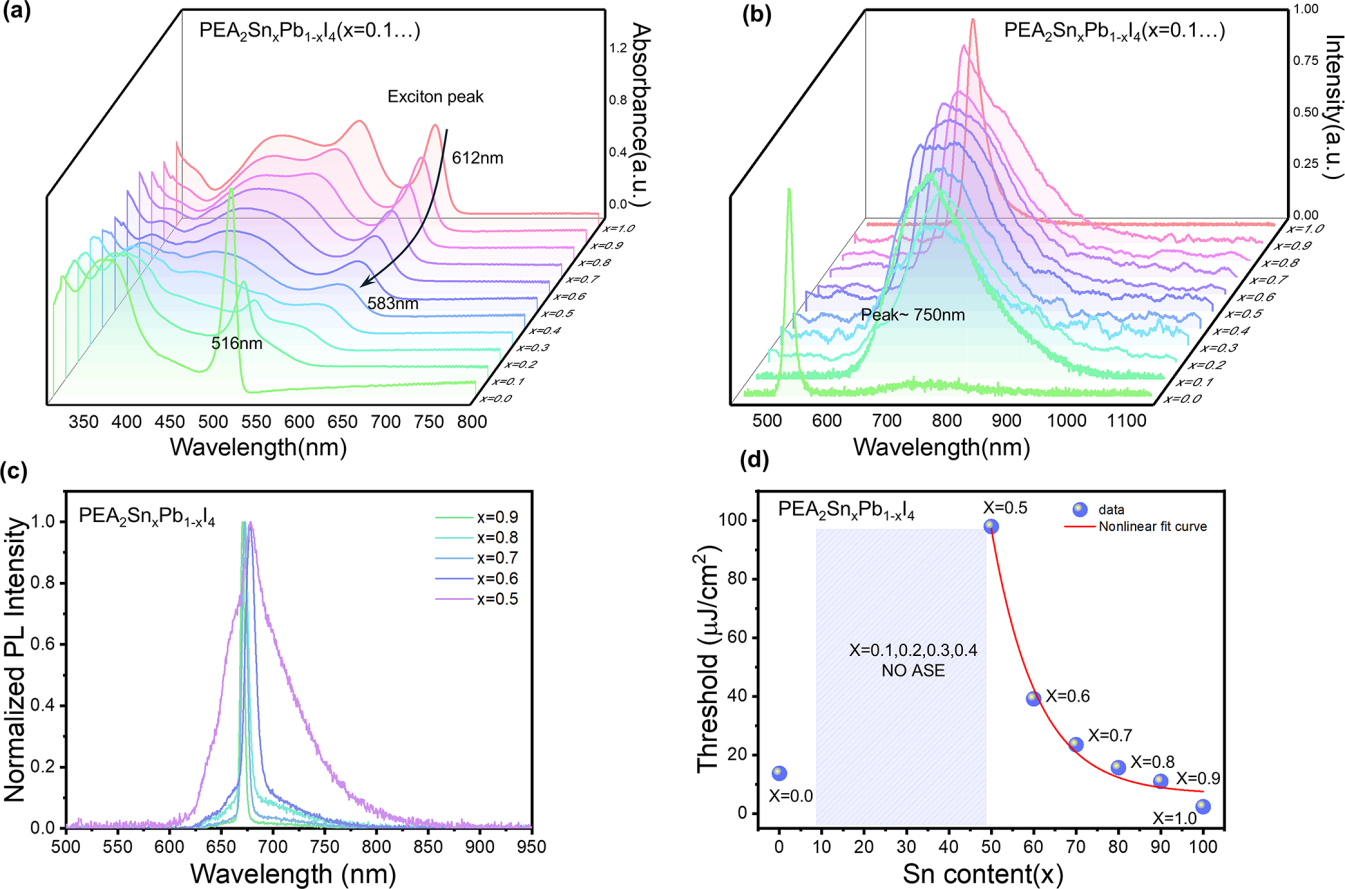
Optical characterization of PEA_2_Sn_x_Pb_1‑x_I_4_ mixed systems. (a) UV–vis absorption and (b) photoluminescence spectra of the PEA_2_Sn_*x*_Pb_1–*x*_I_4_ films, at different mixing ratios (*x* values) at room temperature. (c) ASE spectra (78 K) under 515 nm excitation wavelength and 300 fs pulse width for the compositions in the range *x* = 0.9–0.6, and the composition *x* = 0.5 measured under 400 nm excitation wavelength and 300 fs pulse width. (d) ASE threshold as a function of the Sn content (*x*) (78 K). No ASE is observed in the range of Sn content (*x* = 0.1–0.4).

Lead introduction in the tin perovskite has a deep impact on the photoluminescence, leading to the rise of a broadband luminescence (fwhm ∼ 130 nm) around 750 nm (Figure 4b). We note that this broadband emission is also present in the pure PEA_2_PbI_4_, but with much lower intensity compared to the excitonic luminescence. This red emission has been previously attributed to surface defect states in pure PEA_2_PbI_4_,^47^ and to the combination of I vacancies-induced sub-bandgap states as well as charge transfer states between the iodide and the tin-rich phase in mixed stoichiometries.^48^ In this regard, controlling the Sn/Pb ratio allows for modulating the defectivity of the material and can be used as a case study of the impact of defects on the optoelectronic properties. Local structural changes in the coordination of the metal cation were indeed observed upon Sn/Pb mixing via ssNMR with a clear impact on the asymmetry parameter η. With greater lead contents in PEA_2_Sn_*x*_Pb_1–*x*_I_4_, the defect density increases with a consequent gradual increase in the ASE threshold from 11 up to 98 μJ/cm^2^ (at 78 K) as the Sn content decreased from *x* = 0.9 to *x* = 0.5 (Figure 4c,d). For higher lead contents, ASE cannot be achieved, highlighting the detrimental role of these defect states. Given that broadband emission is also observable in the pure PEA_2_PbI_4_ (although with a weaker contribution than in the mixed compositions), we believe that defects may represent another significant loss channel hampering population inversion in PEA_2_PbI_4_, as previously demonstrated also in other defective materials like BA_2_SnI_4_,^19^ which adds to the effects caused by Auger recombination previously discussed. This makes the composition a critical step in PEA_2_PbI_4;_ even concerning the (PEA)I content,^49^ we find that an excess of 10–30% over the stoichiometric amount shall be used in order to observe ASE; conversely, this has nearly no effect on PEA_2_SnI_4_ (Figure S22).

Finally, we investigated the properties of the pure compounds at higher temperatures. Interestingly, the photoluminescence (PL) lifetime of the spontaneous emission has an opposite trend between the tin and lead compounds (Figure 5a,b). As the temperature increases from 78 to 293 K, the PL lifetime of PEA_2_PbI_4_ decreases (Table S2), which can be attributed to the enhanced probability of nonradiative decay facilitated by phonon scattering at elevated temperatures, a behavior commonly observed in semiconductors (Figure 5b). On the contrary, PEA_2_SnI_4_ showed an atypical trend where its PL lifetime is increased with increasing temperatures (Figure 5a and Table S2). We have previously attributed this peculiar trend to the presence of dark excitonic states located 10 meV below the lowest radiative exciton;^37^ these can act as a reservoir for the excited state population, favoring radiative recombination at high temperature. Under near-bandgap fs pumping, we could probe ASE up to 293 K only for PEA_2_SnI_4_ (threshold fluence is 226 μJ/cm^2^ which corresponds to the excitation density of 1.84 × 10^19^ cm^–3^), while ASE ceases beyond 118 K in PEA_2_PbI_4_ (threshold fluence is 46 μJ/cm^2^ which corresponds to the excitation density of 3.98 × 10^18^ cm^–3^) (Figure 5c–e), which maintains stable for 10s of seconds at their highest corresponding working temperatures (Figure S25).

**Figure 5:**
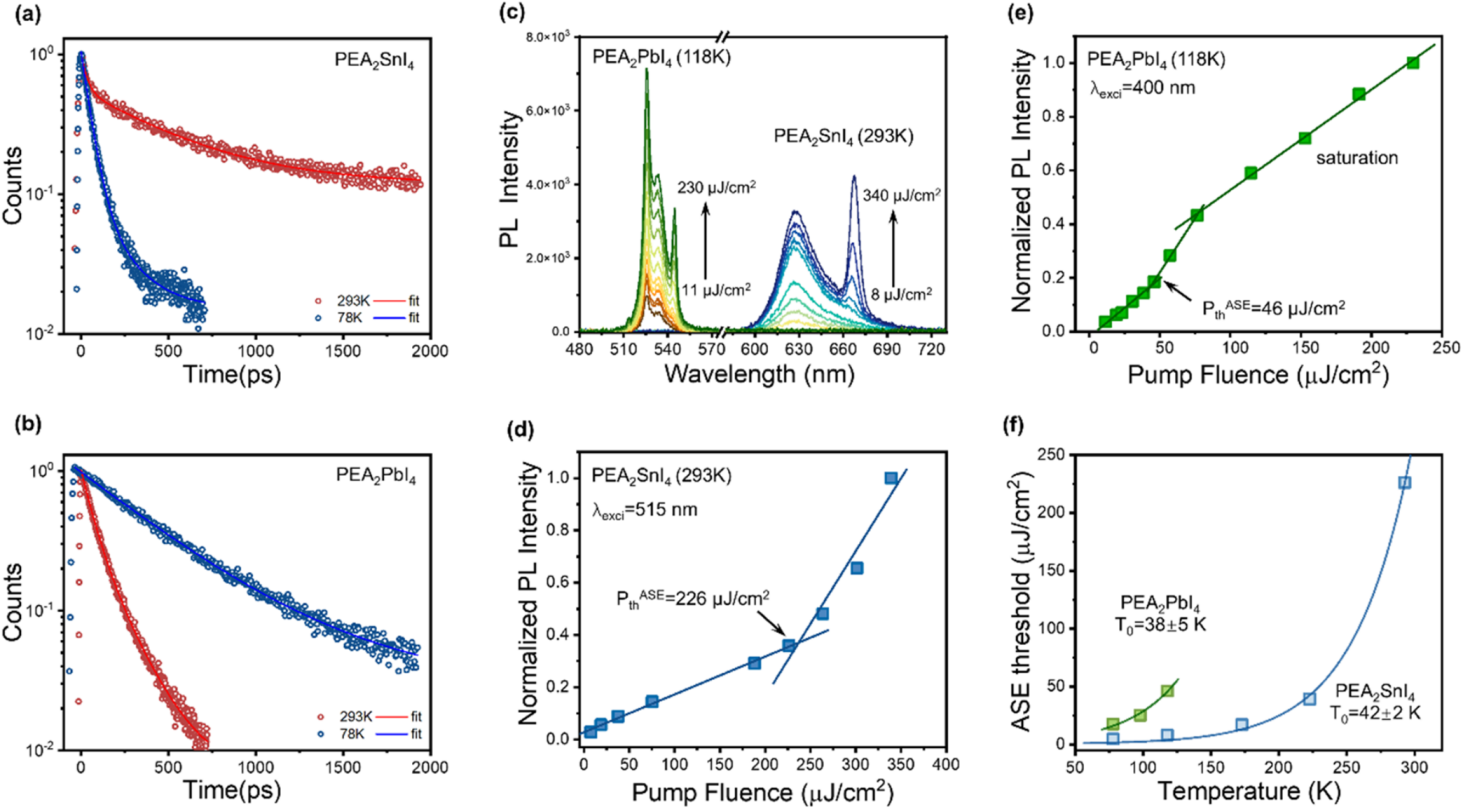
Time-resolved photoluminescence (TRPL) and ASE temperature dependence. TRPL decay dynamics at 293 K (red) and 78 K (blue) of PEA_2_SnI_4_ (a) and PEA_2_PbI_4_ (b). (c) Fluence-dependent PL spectra of PEA_2_SnI_4_ at 293 K with pump fluence increasing from 8 to 340 μJ/cm^2^ (pulse width 300 fs, λ_exci_ = 515 nm, repetition rate 2 kHz), and of PEA_2_PbI_4_ at 118 K with pump fluence rising from 11 to 230 μJ/cm^2^ (pulse width 300 fs, λ_exci_ = 400 nm, repetition rate 2 kHz). ASE intensity evolution as a function of the excitation fluence at the highest working temperature for (d) PEA_2_SnI_4_ at 293 K and (e) PEA_2_PbI_4_ at 118 K. (f) ASE threshold evolution versus temperature; the experimental data were fitted using the exponential function $F_{\mathrm{th}}=F_{0}\times \exp\left(\frac{T}{T_{0}}\right)$ , where *F*_th_ is the threshold fluence, *F*_0_ is the threshold fluence at 0 K, and *T*_0_ is the characteristic temperature, which results to be 42 ± 2 K for PEA_2_SnI_4_ and 38 ± 5 K for PEA_2_PbI_4_.

The ASE threshold undergoes an exponential increase with temperature, which can be fitted with the function *F*_th_ = *F*_0_·exp­(*T*/*T*_0_), where *F*_0_ is the threshold fluence at 0 K and *T*_0_ represents the characteristic temperature. The *T*_0_ is around 40 K for both materials (Figures 5f, S23 and S24), which is low compared to that of fully inorganic semiconductors, where it typically surpasses 100 K.^50^ This indicates a strong effect of temperature on the ASE quenching, which can be related to the motion of the ethylammonium moiety previously discussed through ssNMR, despite the increased rigidity provided by the aromatic ring. In fact, due to the close interaction via hydrogen bonding with the metal halide framework, the motion of the alkylammonium moiety may still exert a strong influence on the inorganic lattice. The early disappearance of ASE should not be attributed to a sudden structural change of PEA_2_PbI_4_ due as there is no evident shift in the ^13^C ssNMR signals upon cooling (Figure 1d). However, the significant structural rigidification observed at low temperature (in particular of the ethyl group) can alleviate nonradiative recombination due to phonon scattering, thus enhancing the luminescence properties.

Based on our analysis, we attribute the inferior ASE performance of the PEA_2_PbI_4_ to a combination of factors, which include low excitation density offset for Auger recombination, faster PL decay dynamics at high temperature, and carrier trapping at defect states. This is in contrast with its tin counterpart, where the smaller impact of Auger recombination, defects trapping, and longer PL lifetime at room temperature help to counterbalance the nonradiative losses due to thermal vibrations of the crystal lattice, allowing ASE to be observed at higher temperatures up to 293 K.

## Conclusion

This study provides a thorough analysis of the structural, optical, and ASE properties of PEA_2_PbI_4_ and PEA_2_SnI_4_ thin films. Our findings demonstrate the achievement of stable ASE at 78 K in both the lead and tin perovskite with thresholds of 17 and 5 μJ/cm^2^, respectively. However, the ASE quenches above 120 K in PEA_2_PbI_4_, while it remains stable up to room temperature in PEA_2_SnI_4_ with a threshold of 226 μJ/cm^2^. Shorter laser pulse width (fs) and near band gap excitation are critical to lower the threshold and extend ASE stability toward higher temperatures. We attribute the lower optical gain in PEA_2_PbI_4_, relative to its tin counterpart, primarily to three factors: pronounced Auger recombination (with an onset carrier density nearly an order of magnitude lower than in the tin perovskite), accelerated excited-state decay at elevated temperatures, and trap-mediated recombination. Aromatic cations like PEA can yield enhanced rigidity to the crystal structure, which is beneficial in eliminating nonradiative recombination pathways,^19^ but the motion of the ethylammonium linker near the inorganic framework may still be associated with the rapid exponential growth of ASE threshold with temperature in both compounds, leading to severe ASE thermal quenching. We believe that defect passivation strategies and molecular engineering of the templating cations will need to be developed to further improve the optical gain properties of 2D perovskites for application as advanced coherent light sources.

## Experimental Section

### Perovskite Synthesis

For the synthesis of PEA_2_SnI_4_ and PEA_2_PbI_4_ thin films used for optical and morphological characterizations, the organic precursors (PEA)I were mixed with SnI_2_ and PbI_2_ in a 2:1 molar ratio in dimethylformamide (DMF), to obtain a solution with a concentration of 0.2 M. The mixture was heated at 100 °C for 1 h and then filtered (PTFE filters, 0.45 μm). Fused silica substrates were cleaned by sonication in deionized water, acetone, and isopropanol, followed by oxygen plasma treatment for 10 min. The solution was dropped on the substrate and spin-coated at 5000 rpm for 30 s. The thin films were annealed at 100 °C for 10 min. The sample preparation and storage were completely done in a nitrogen-filled glovebox. The mixed perovskite PEA_2_Sn_*x*_Pb_1–*x*_I_4_ films were tuned by the volume ratio of precursor PEA_2_SnI_4_ and PEA_2_PbI_4_ solutions with the same molar concentration. Optimization was achieved by incorporating an excess of (PEA)­I, ranging from 10% to 50% beyond the stoichiometric amount.

Crystals of PEA_2_Sn_*x*_Pb_1–*x*_I_4_ (used for ssNMR characterization) were prepared by the following procedure. For PEA_2_PbI_4_, PbI_2_ (461 mg, 1 mmol) was dissolved in 1.5 mL of HI (prepared by mixing 1.5 mL of HI (57 wt %) with 1 mL of H_3_PO_2_ (50 wt %)). Separately, 92.4 μL phenethylamine (PEA) was neutralized with 1 mL of HI solution (prepared by mixing 1.5 mL of HI (57 wt %) with 1 mL of H_3_PO_2_ (50 wt %)), causing the precipitation of a white solid, which was redissolved upon heating at 100 °C for 10 min. This was then added to the PbI_2_ solution prepared previously, and the mixture was heated at 130 °C for 20 min until it became a clear yellow solution under magnetic stirring on a hot plate. After that, the stirring was stopped and the solution was left to cool down to room temperature over 20 h, to obtain the precipitate of PEA_2_PbI_4_ crystals. The microcrystals were collected by filtration and dried under vacuum, which was used for further studies. The PEA_2_SnI_4_ crystals were synthesized by the same procedure as before, except SnI_2_ (372.5 mg, 1 mmol) was used in place of PbI_2_. PEA_2_Sn_0.5_Pb_0.5_I_4_ was synthesized by employing a 1:1 stoichiometric ratio of PbI_2_ and SnI_2_ and following the same above procedure.

### Solid-State Nuclear Magnetic Resonance (ssNMR)

Solid-state NMR measurements were performed on a Bruker Avance NEO WB with the wide-bore magnet (89 mm) operating at a ^1^H frequency of 400 MHz. The chemical shift was calibrated with reference to the ^13^C signal of adamantane at 37.77 ppm.

A 1.9 mm cross-polarization-magic angle spinning (CP-MAS) probe with spinning frequency up to 42 kHz was used for the characterization, employing 1.9 mm zirconia rotors with VESPEL turbines. A one-dimensional ^13^C­{^1^H} spectrum was obtained using cross-polarization, where magnetization was first excited in the ^1^H nuclei and then transferred to the targeted heteronuclear. During data acquisition, heteronuclear decoupling was applied using the SPINAL64 sequence. For ^13^C measurements, the spin rotation frequency was set to 12 kHz, and for ^1^H to 42 kHz for all samples. The contact time for CP was set to 1.5 ms for PEA_2_SnI_4_ and 4.5 ms for PEA_2_PbSnI_4_, PEA_2_SnI_4_. The 90° pulse durations were 1.5 μs for ^13^C and 2.27 μs for ^1^H. The ^13^C spin–lattice relaxation *T*_1_ at temperatures in the range of 260 and 330 K was measured with the TORCHIA pulse sequence under MAS at 12 kHz; for ^1^H, inversion recovery pulse sequence applied at 42 kHz. Temperature calibration of the sample under magic angle spinning conditions was performed by measuring the spin–lattice relaxation time (*T*_1_) of ^79^Br in KBr via an inversion recovery experiment. ^207^Pb and ^119^Sn spectra were acquired with a Hahn-echo pulse sequence with a 90° pulse duration of 2.4 μs, echo delay of 12.4 μs, and accumulation of 500,000 transients. Lineshape analysis and determination of isotropic chemical shift (δ_iso_), chemical shift anisotropy (Δδ), and asymmetry index (η) were performed with the SOLA software package implemented in TopSpin.

Low-temperature measurements were carried out using a 3.2 mm low-temperature magic angle spinning (LT-MAS) probe operating at spinning rates up to 24 kHz, with 3.2 mm zirconia rotors and turbines. The probe temperature was controlled by the LT-MAS cooling cabinet, the system enables sample cooling down to 80 K. For ^13^C measurements, the contact time for CP was set to 1.9 and 4.5 ms for PEA_2_PbI_4_ at 96 and 307 K, respectively.

### Temperature-Dependent UV–vis Spectroscopy

Steady-state absorption spectra were measured on 2D perovskite thin films deposited on quartz glass using a UV/vis/NIR spectrophotometer, Lambda 1050, PerkinElmer, in the wavelength range 300–800 nm (4.13–1.55 eV), a step size of 1.0 nm, and a slow scan speed. A cryostat (Oxford Instruments) was used to perform cryogenic measurements between 78 K and room temperature.

### Optical Characterization

Two different pulsed lasers are used for the measurements. The picosecond pulsed laser (Innolas Picolo) has a pulse duration of 800 ps and a tunable repetition rate of single-shot 10 kHz. Its second (532 nm) and third harmonic (343 nm) are used for the long pulse width measurements. The femtosecond pulsed laser (Light Conversion Pharos) has a pulse duration of 300 fs and a repetition rate of single-shot 500 kHz. The femtosecond laser is coupled to a harmonic generator (HIRO) whose second (515 nm) and third harmonics (343 nm) are used for the short pulse width measurements. In addition, the laser system is also coupled to a collinear optical parametric amplifier (Orpheus) with a tunable wavelength range of 315–2600 nm.

For the ASE measurements, the laser signal was focused on the sample with a 10 cm spherical lens, and the diameter of the beam size is around 120–130 μm, then collected with a Maya 2000 Pro visible spectrometer and a 550 nm long pass filter and a 450 nm long pass filter. Moreover, to describe the behavior of the photoluminescence spectra as a function of excitation fluence, the intensities and line width were extracted as follows: (1) below threshold, the emission closest to the ASE peak was fitted to a Gaussian function, and (2) above threshold, the ASE peak was fitted to a Lorentzian curve. The fluence-dependent PL measurements were performed under the same configuration.

### Time-Resolved Photoluminescence

TRPL measurements were recorded using a Hamamatsu streak camera with a resolution of 2 ps in the 200 ps measurement range and of 100 ps in the 1.5 ns measurement range. The lead sample was excited at 400 nm while the tin sample was excited at 520 nm using the frequency-doubled output of a Chameleon (Coherent) ultrafast oscillator with a pulse duration of 30 fs and an 85 MHz repetition rate.

### Atomic Force Microscopy (AFM) Images

The topography images have been acquired with the Bruker Dimension Icon AFM, employing Oxford Instruments tips optimized for tapping mode and electrical measurements (ASYELEC.01-R2,81 kHz actual resonance frequency). We set up a 2 × 2 μm^2^ scan area, which was scanned at a rate of 1 line per second, with a 512 × 512 pixel resolution. The fast scan rate was made possible by the very low roughness of the samples. The image files were post-treated with Gwyddion: the second-order polynomial background was subtracted, and subsequently, each scan line was flattened by median subtraction.

## Supplementary Material



## Notes

Additional data on XRD, PXRD, SEM, and AFM; details on spin–lattice relaxation time analysis and fitting parameters; temperature-dependent ^13^C spin–lattice relaxation times; temperature-dependent absorption, photoluminescence and ASE of perovskite films; fluence-dependent ASE; ASE under different pulse widths, excitation energies and repetition rates; ASE stability (PDF)
