# Supplementary material for: Role of the Metal Cation on the Amplified Spontaneous Emission Properties of Two-Dimensional Perovskites
Source: J Am Chem Soc. 2025 Nov 20;147(48):44175–84. doi: 10.1021/jacs.5c13296 (PMC12679635; doi:10.1021/jacs.5c13296)
Supplement: Supplementary file 1 [file ja5c13296_si_001.pdf]

Supporting Information

Role of the metal cation on the amplified spontaneous emission properties of two-dimensional perovskites

Yarong He[1,2], E Laine Wong[1], Nurgul Sarsembek[3], Ranita Samanta[3], Davide Regaldo[1], Andrea Pianetti[1], Michela Cecconi[3], Guglielmo Lanzani,[1,2]
Annamaria Petrozza[1,*], Daniele Cortecchia[1,3,*]

[1]*Center for Nano Science and Technology @Polimi, Istituto Italiano di Tecnologia, via Rubattino 81, Milano, 20134 Italy*
[2]*Dipartimento di Fisica, Politecnico di Milano, Milano, 20133 Italy*
[3]*Department of Industrial Chemistry "Toso Montanari", University of Bologna, via Piero Gobetti 85, 40129 Bologna, Italy.*

*Annamaria.Petrozza@iit.it; Daniele.Cortecchia2@unibo.it

Contents

1. **Materials**
2. **Figure S1.** PXRD stack plot of $PEA_2Sn_xPb_{1-x}I_4$ perovskite.
3. **Spin-relaxation analysis**
   Table S1. Fitting parameters of temperature-dependent ^{1}H T_1 measurements for PEA_2XI_4 (X=Pb, Sn).
4. **Figure S2.** Temperature dependence of the ^{13}C spin–lattice relaxation times (T_1) for PEA_2PbI_4 and PEA_2SnI_4.
5. **Figure S3.** Thin film X-ray Diffraction (XRD) and morphology characterization (SEM and AFM) of the PEA_2PbI_4 and PEA_2SnI_4 perovskites.
6. **Figure S4.** Temperature-Dependent photoluminescence and Exciton-phonon coupling strength fitting in PEA_2PbI_4 and PEA_2SnI_4.
7. **Figure S5.** Temperature-Dependent Double-Peak Gaussian Fitting of PL Spectra in PEA_2SnI_4 Thin Films (293–218 K).
8. **Figure S6.** Temperature-Dependent Double-Peak Gaussian Fitting of PL Spectra in PEA_2SnI_4 Thin Films (198–78 K).
9. **Figure S7.** Temperature-Dependent Double-Peak Gaussian Fitting of PL Spectra in PEA_2PbI_4 Thin Films (293–218 K).

10. **Figure S8.** Temperature-Dependent Double-Peak Gaussian Fitting of PL Spectra in PEA_2PbI_4 Thin Films (198–78 K).
11. **Figure S9.** Fluence-Dependent PL Spectra of PEA_2SnI_4 at 78 K under different pulse-width pumps.
12. **Figure S10.**Fluence-Dependent PL Spectra of PEA_2SnI_4 and PEA_2PbI_4 78 K under the same pump conditions.
13. **Figure S11.** Fluence-Dependent PL Spectra of PEA_2PbI_4 at 78 K under different pulse width pumps.
14. **Figure S12.** Schematic of a three-level model.
15. **Figure S13.** ASE threshold comparison between the Sn and Pb under the different repetition rates of pumps at 78 K.
16. **Figure S14.** Fluence-Dependent PL Spectra of PEA_2SnI_4 and PEA_2PbI_4 at 78 K from 10 kHz to 500 kHz.
17. **Figure S15.** Statistical data of ASE threshold at low temperature of Sn (sixteen samples) and Pb (twenty-five samples).
18. **Figure S16.** Temperature-Dependent Absorption and Exciton Binding Energy in PEA_2SnI_4 and PEA_2PbI_4.
19. **Figure S17.** Relative Photoluminescence Quantum Yield (PLQY) of PEA_2SnI_4 and PEA_2PbI_4 at room temperature.
20. **Figure S18.** X-ray diffraction (XRD) of $PEA_2Sn_xPb_{1-x}I_4$(x=0.1...) polycrystalline films.
21. **Figure S19.** The picture of samples in $PEA_2Sn_xPb_{1-x}I_4$(x=0.1...) polycrystalline films.
22. **Figure S20.** Scanning electron microscope (SEM) images with the same magnification (30 kx) of $PEA_2Sn_xPb_{1-x}I_4$ (x=0.1…) polycrystalline films.
23. **Figure S21.** Atomic force microscopy (AFM) images (2 μm×2 μm) of Sn(x=0.9), Sn(x=0.5), and Sn(x=0.1) from the $PEA_2Sn_xPb_{1-x}I_4$ (x=0.1…) polycrystalline films.
24. **Figure S22.** ASE threshold of PEA_2SnI_4 and PEA_2PbI_4 as a function of the (PEA)I excess treatment.
25. **Figure S23.** Fluence-dependent PL Spectra of PEA_2SnI_4 at different temperatures under the femtosecond excitation.
26. **Table S2**. Bi-exponential decay parameters, and fast and slow delay of TRPL analysis of Sn and Pb at 293 K and 78 K

27. **Figure S24.** Pump Fluence-Dependent Photoluminescence and ASE Threshold Behavior of PEA_2PbI_4 Thin Films at 98 K and 118 K under 400 nm Femtosecond Excitation
28. **Figure S25.** ASE Spectral Evolution and Stability of PEA_2SnI_4 and PEA_2PbI_4 Thin Films under Continuous Femtosecond Laser Excitation at 293 K and 78 K

Materials

N,N-Dimethylformamide (DFM, anhydrous, 99.8%), HI (57 wt. % in H_2O stabilized), H_3PO_2 were purchased from Sigma-Adrich. Phenethylammonium iodide (PEAI, CAS 151059-43-7) was purchased from Greatcell Solar. Lead (II) iodide (PbI_2, 99.99%, CAS 10101-63-0) and tin (II) iodide (SnI_2, for perovskite precursor) were purchased from Tokyo Chemical Industry (TCI).

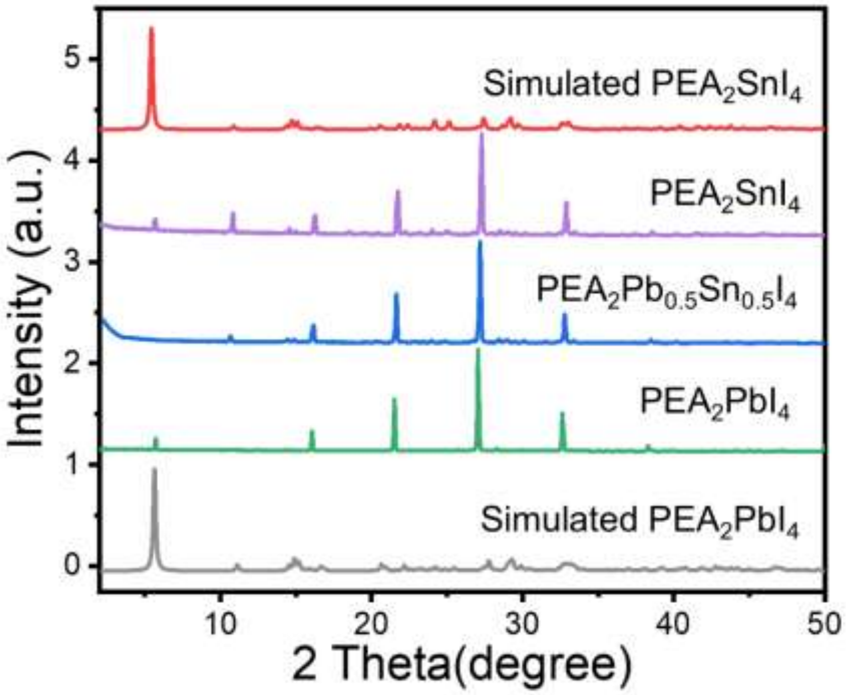

Figure S1: PXRD stack plot of $PEA_2Sn_xPb_{1-x}I_4$ perovskite. The PXRD patterns of the synthesized powders of $(PEA)_2PbI_4$ and $(PEA)_2SnI_4$ show a strong preferential orientation and are consistent with their reported crystal system *P*1and *P*-1, respectively[1,2].Also $(PEA)_2Pb_{0.5}Sn_{0.5}I_4$ maintained a similar diffraction pattern due to the nearby value of ionic radii of Pb^{2+} and Sn^{2+} ($\mathbf{r}_{Pb(II)}$: 120 pm and $\mathbf{r}_{Sn(II)}$: 93 pm). The simulated XRD patterns (red and grey lines) were calculated from the previously published single crystal structures with the software Mercury [1,2].

Spin-relaxation analysis

The temperature dependence of the ^1H spin–lattice relaxation times (T_1) was analyzed within the framework of the Bloembergen–Purcell–Pound (BPP) model[3], which describes nuclear spin relaxation driven by dipole–dipole interactions modulated by molecular motions. In this model, the relaxation rate is expressed as:

$$\frac{1}{T_1} = C\left[\frac{\tau_c}{1+\omega^2 \tau_c{}^2} + \frac{2\tau_c}{1+4\omega^2 \tau_c{}^2}\right] \quad (1)$$

where, $C = \frac{3\mu_0^2 \hbar^2 \gamma^4}{160\pi^2 r^6}$ is a constant independent of correlation time and magnetic field strength, μ_0 is the vacuum permeability, ħ is the reduced Planck constant, γ is the gyromagnetic ratio of the proton, and ω_0 is the Larmor frequency. τ_c is the correlation time of the reorientational motion, and r is the internuclear separation between coupled protons. Assuming thermal activations, the temperature dependence τ_c represented by Arrhenius equation[4]:

$$\tau_c = \tau_0 * e^{\frac{Ea}{RT}} \quad (2)$$

where, τ_0 is a pre-exponential factor, T is the temperature, R is the gas constant, and Ea activation energy. Activation parameters for molecular motion were extracted from the slopes of the linear fits by applying (1) and (2) on the T_1 data shown in **Figure 1e** and summarized in **Table S1.**

Table S1. Activation parameters determined from ^{1}H T_1 for PEA_2XI_4 (X=Pb, Sn)

Sample	τ_0 (ps)	Ea (kJ/mol)
PEA_2PbI_4	0.57+0.35	11.05±1.78
PEA_2SnI_4	5.48±2.03	6.12±1.89

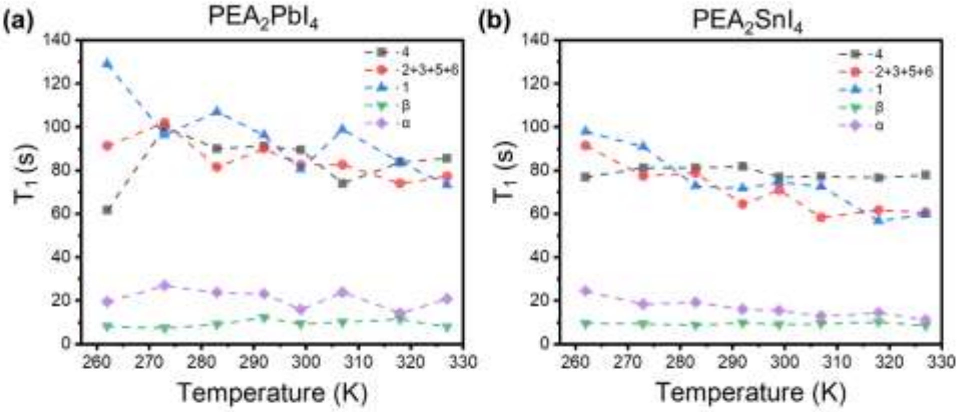

Figure S2. Temperature dependence of the ^{13}C spin–lattice relaxation times (T_1) for (a) PEA_2PbI_4 and (b) PEA_2SnI_4, measured under MAS at 12 kHz over the temperature range of 260–330 K. Different carbon sites within the phenylethylammonium (PEA) cation are labelled according to the numbering scheme in Figure 1: aromatic carbons (1-6), alkyl chain carbons (α, β).

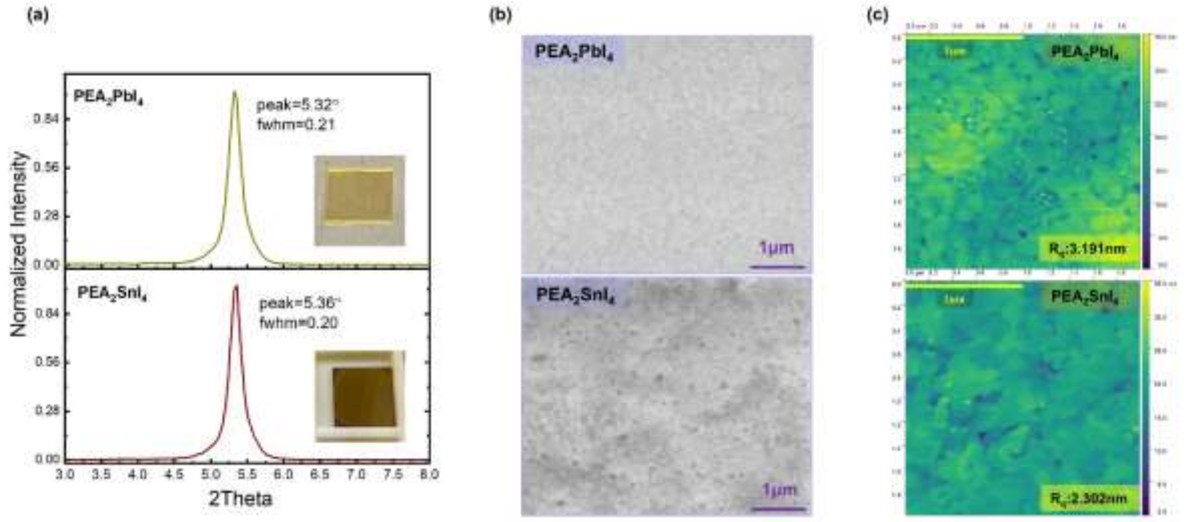

Figure S3 (a). Thin film X-ray Diffraction (XRD) of the PEA_2PbI_4 and PEA_2SnI_4 perovskites. A single diffraction peak for each material is visible due to the strong preferential orientation on the glass substrate. (b). Scanning electron microscope (SEM) images of PEA_2PbI_4 and PEA_2SnI_4 polycrystalline film. (c). Atomic force microscopy (AFM) images (2 μm×2 μm) of PEA_2PbI_4 and PEA_2SnI_4 polycrystalline films on the glass substrate.

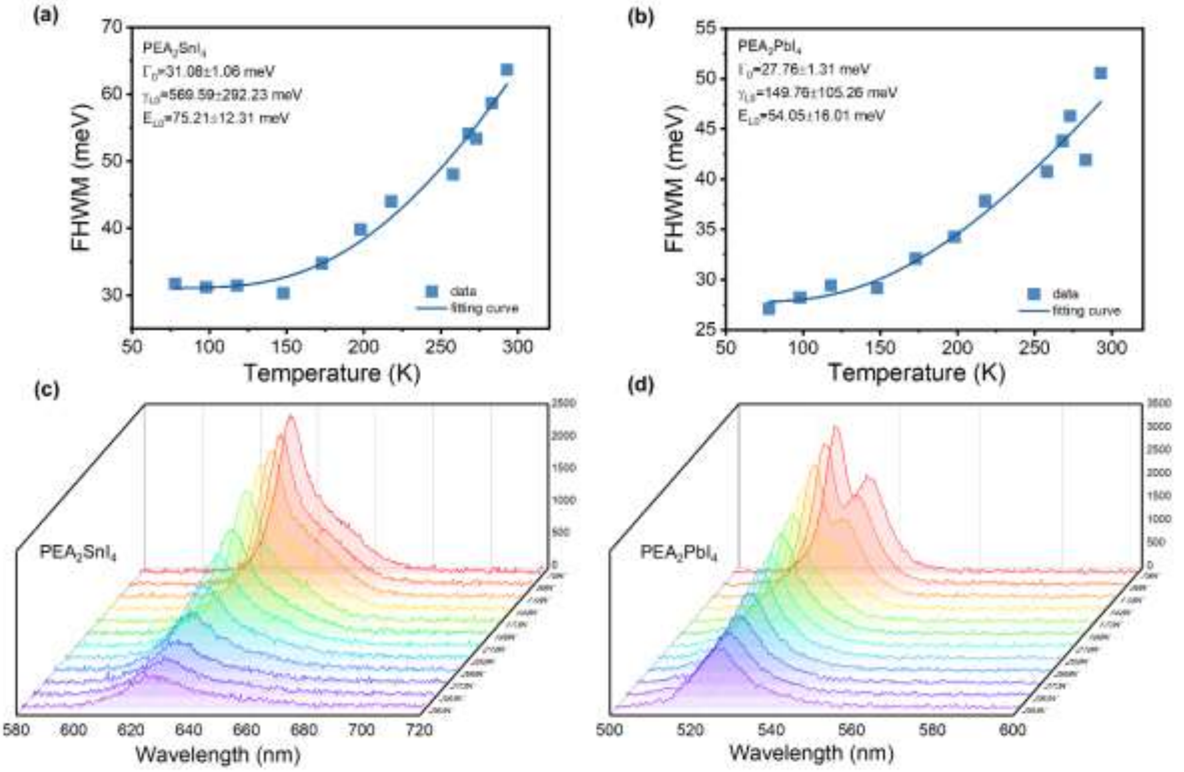

Figure S4 (a). Full width at half maximum of the most intense PL peak as a function of temperature in PEA_2SnI_4 thin film; (b)in PEA_2PbI_4 thin film. The fitting formula of the independent boson population mode[3] is: $\Gamma(T) = \Gamma_0 + \gamma_{L0}\left(\frac{1}{e^{\frac{E_{LO}}{k_B T}}-1}\right)$, where Γ_0 is the temperature-independent peak width, γ_{L0} is the exciton-phonon coupling strength, and E_{L0} is the phonon energy. (c)(d). Temperature-dependent PL spectra of Sn and Pb samples.

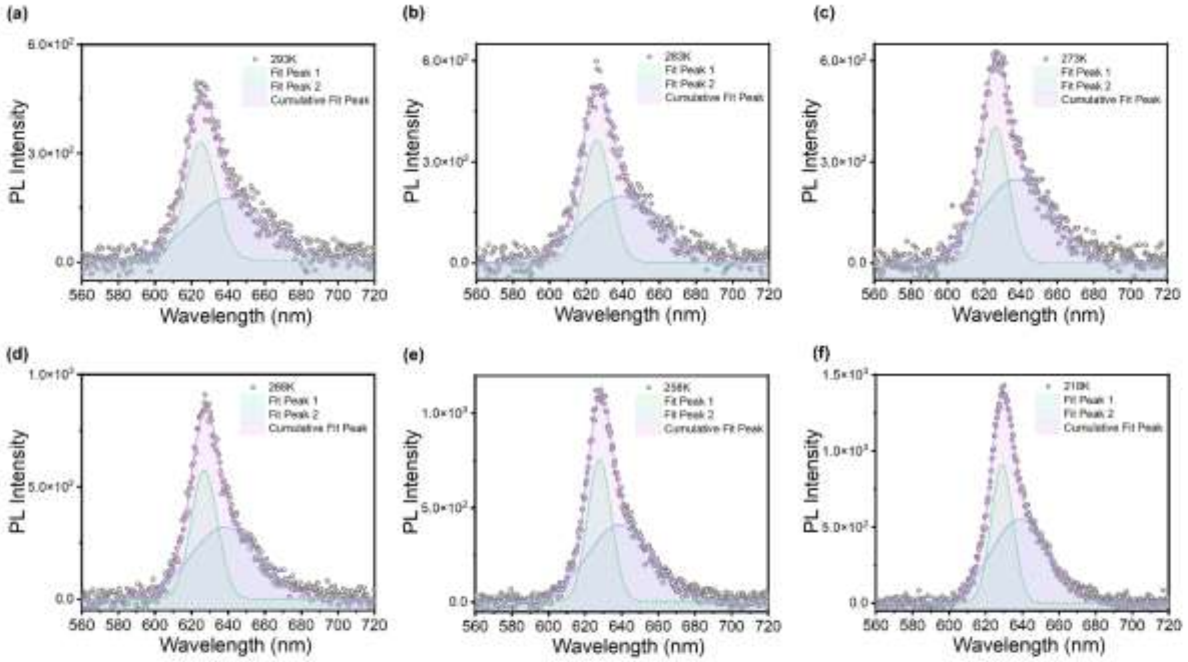

Figure S5 (a)-(f). The double peak fit from the PL spectra data of PEA_2SnI_4 used the Gauss function with the temperature change from 293 K to 218 K.

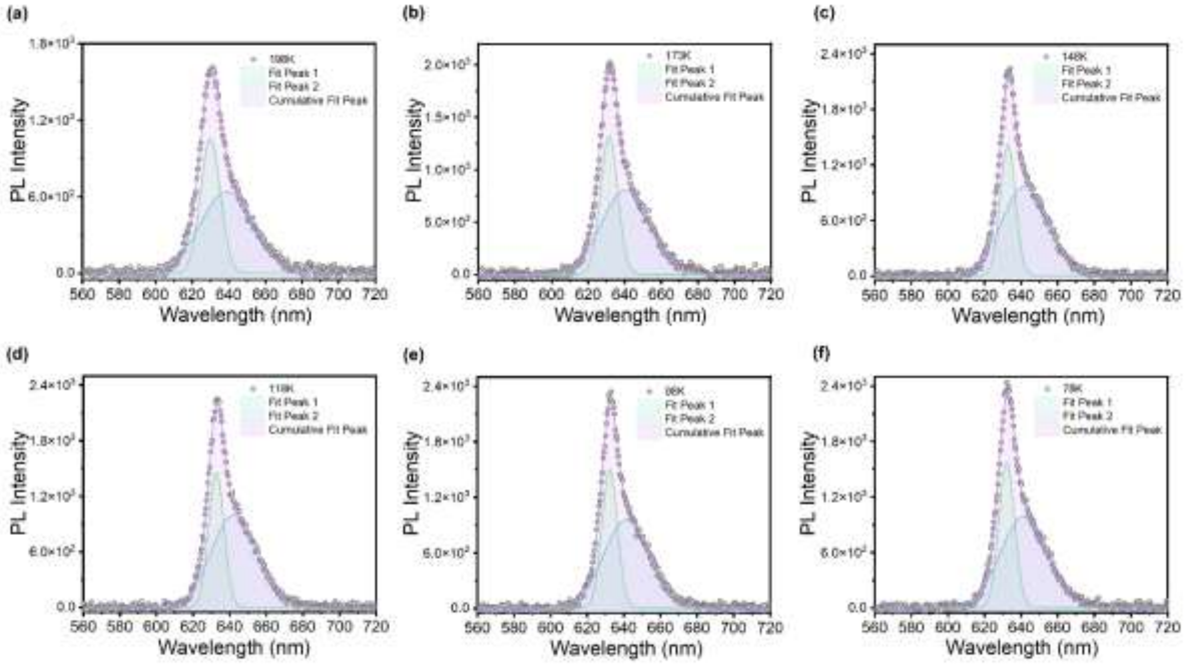

Figure S6 (a)-(f). The double peak fit from the PL spectra data of PEA_2SnI_4 used the Gauss function with the temperature change from 198 K to 78 K.

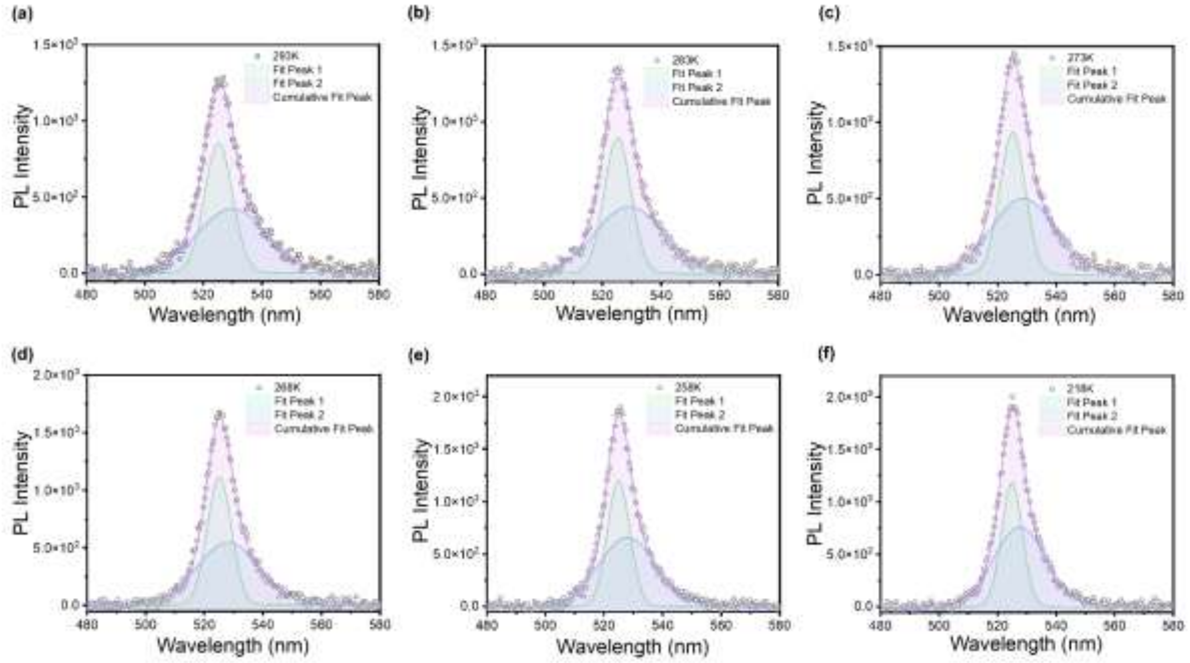

Figure S7 (a)-(f). The double peak fit from the PL spectra data of PEA_2PbI_4 used the Gauss function with the temperature change from 293 K to 218 K.

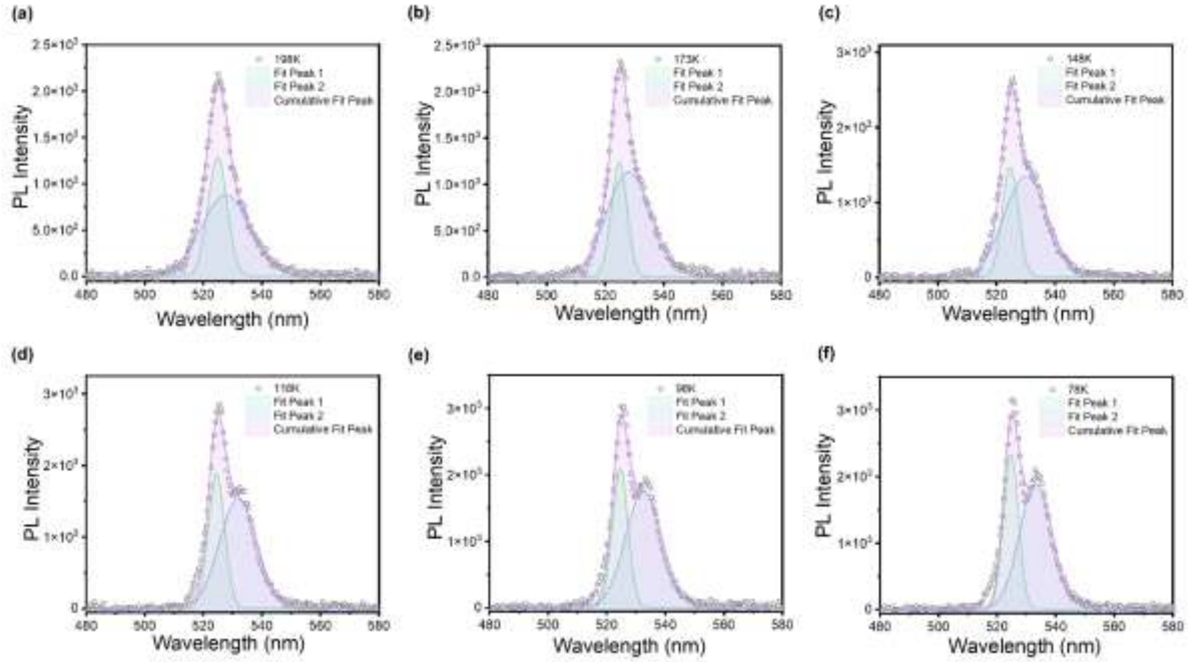

Figure S8 (a)-(f). The double peak fit from the PL spectra data of PEA_2PbI_4 used the Gauss function with the temperature change from 198K to 78 K.

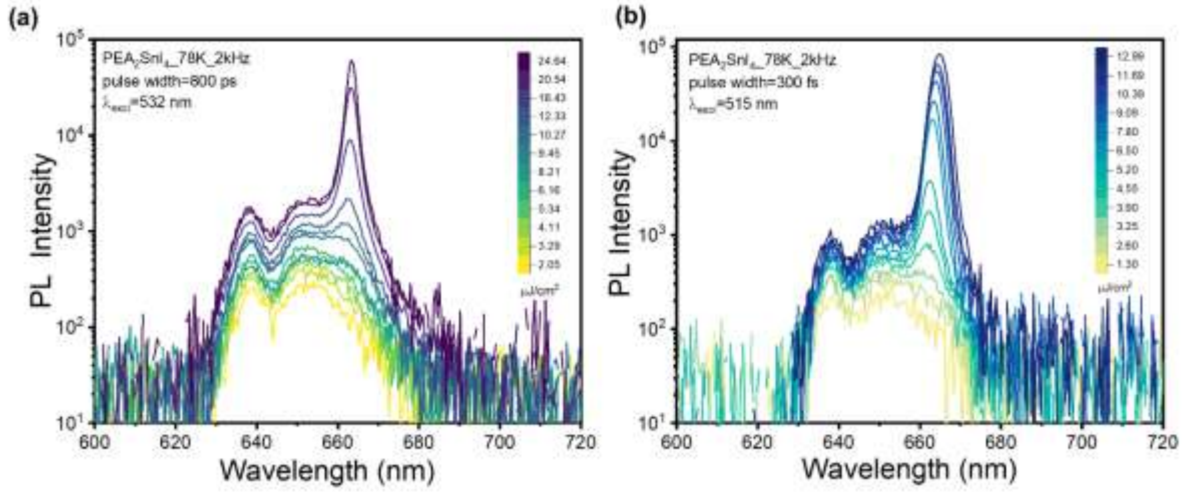

Figure S9 (a). Pump fluence-dependent PL Spectra of PEA_2SnI_4 at 78 K (λ_{exci}=532 nm, repetition rate 2 kHz, pulse width 800 ps). (b) λ_{exci}=515 nm, repetition rate 2 kHz, pulse width 300 fs.

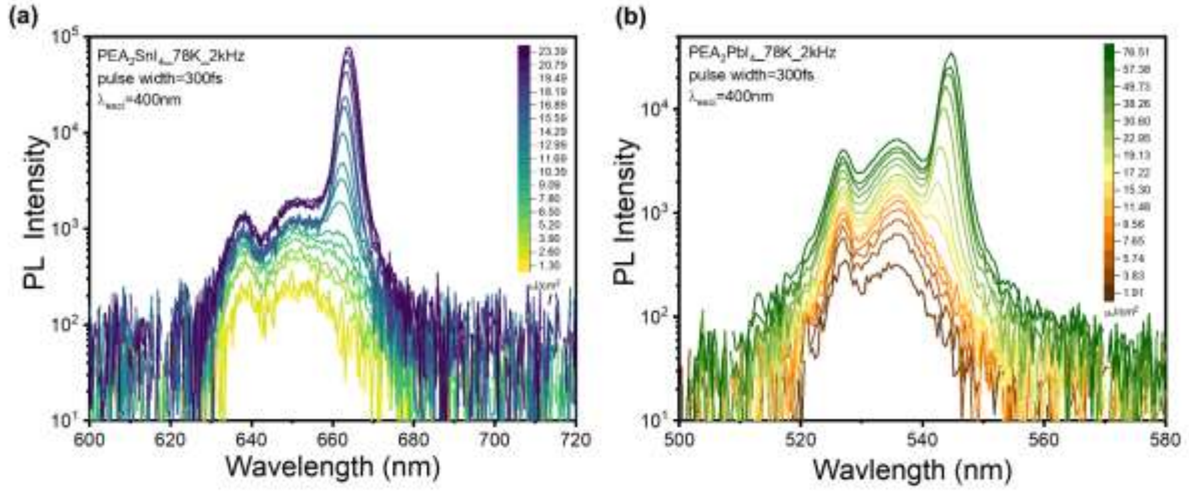

Figure S10 (a). Fluence-dependent PL spectra of PEA_2SnI_4 with fluence intensity increasing from 1.30 to 23.39 μJ/cm^2 at 78 K. (b) Fluence-dependent PL spectra of PEA_2PbI_4 with fluence intensity increasing from 1.91 to 76.51 μJ/cm^2 at 78 K.

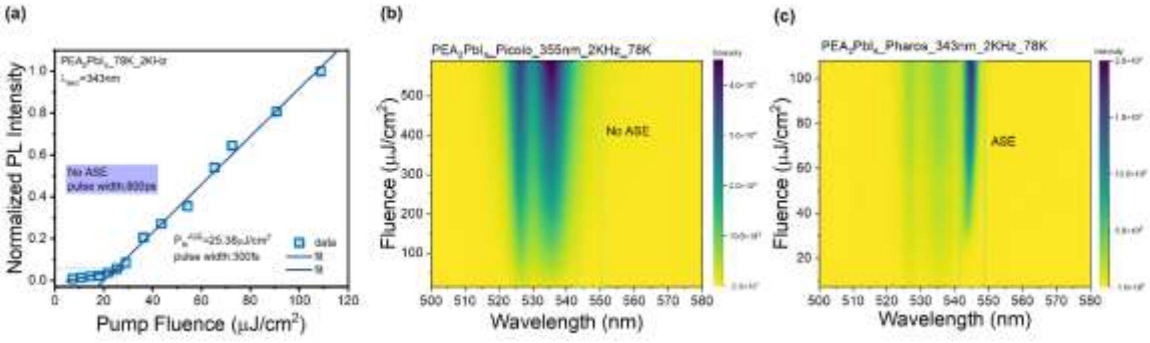

Figure S11 (a). The PL intensity as a function of excitation fluence with a femtosecond pulse laser of Pb sample (green square represents the parameter is pulse duration 300 fs, λexci=400 nm, 2 kHz), but without a picosecond pulse laser. (b). PL spectroscopy of the PEA_2PbI_4 with the picosecond pulse laser as the pumping power increased from 15 $\mu J/cm^2$ to 589 $\mu J/cm^2$ at low temperature. (c). PL spectroscopy of the PEA_2PbI_4 with the femtosecond pulse laser as the pumping power increased from $\mu J/cm^2$ to 109 $\mu J/cm^2$ at low temperature. The ASE can't be observed with the picosecond pump condition in the Pb film.

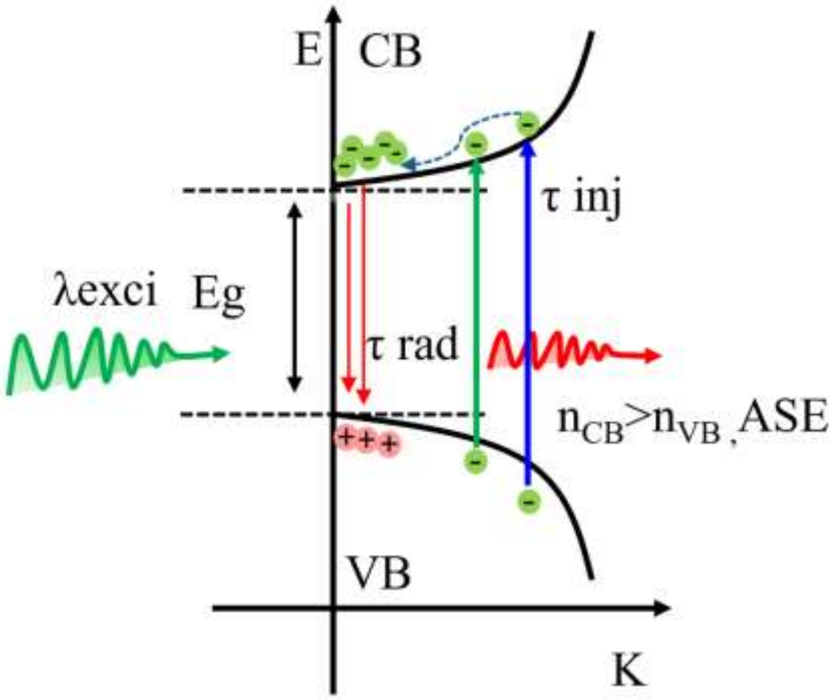

Figure S12. Scheme showing the three-level model, the carrier is excited to the excited state, then the photocarrier quickly relaxes to the band edge in a short time, and then accumulates at the band edge to the population inversion.

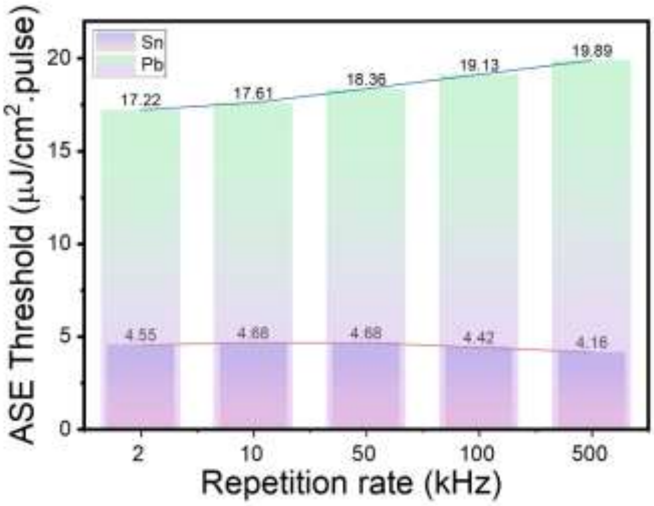

Figure S13. One plot under the different repetition rates of Sn and Pb at 78 K (these threshold values are adopted from the lowest values).

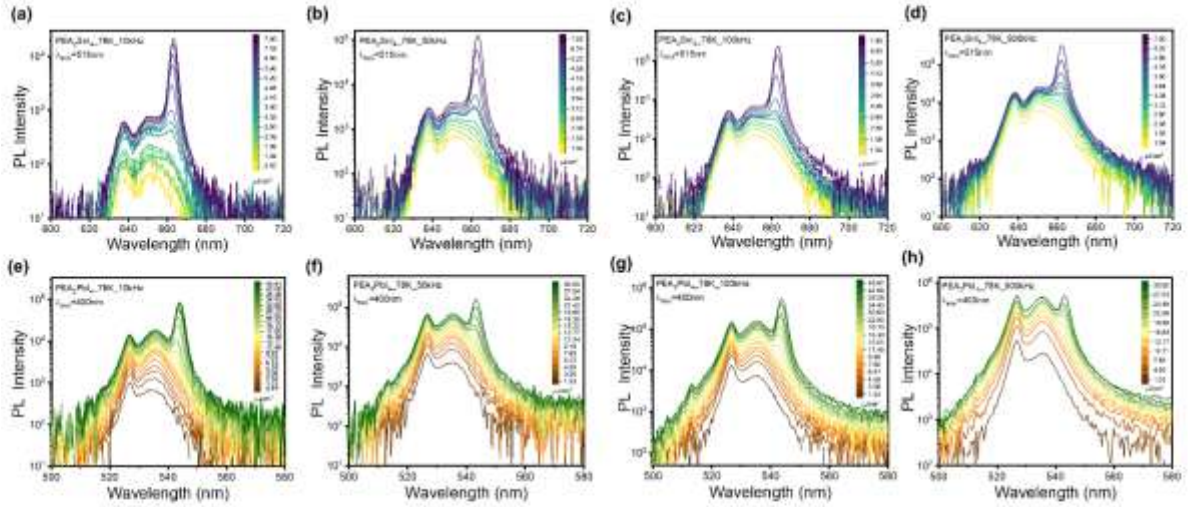

Figure S14 (a)(b)(c)(d). At low temperature, keep the same pump condition as 2 kHz repetition rate (λ_{exci}=515nm and pulse width 300 fs), pump fluence-dependent PL Spectra of PEA_2SnI_4 with the repetition rates changes from 10 kHz to 500 kHz; (e)(f)(g)(h). The same pump condition as 2 kHz repetition rate (λ_{exci}=400 nm and pulse width 300 fs), pump fluence-dependent PL Spectra of PEA_2PbI_4 with the repetition rates changing from 10 kHz to 500 kHz.

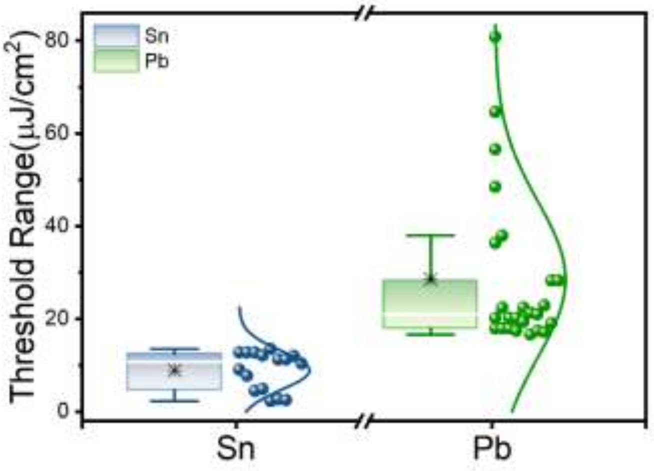

Figure S15. Statistical data of ASE threshold at low temperature of Sn (sixteen samples) and Pb (twenty-five samples).

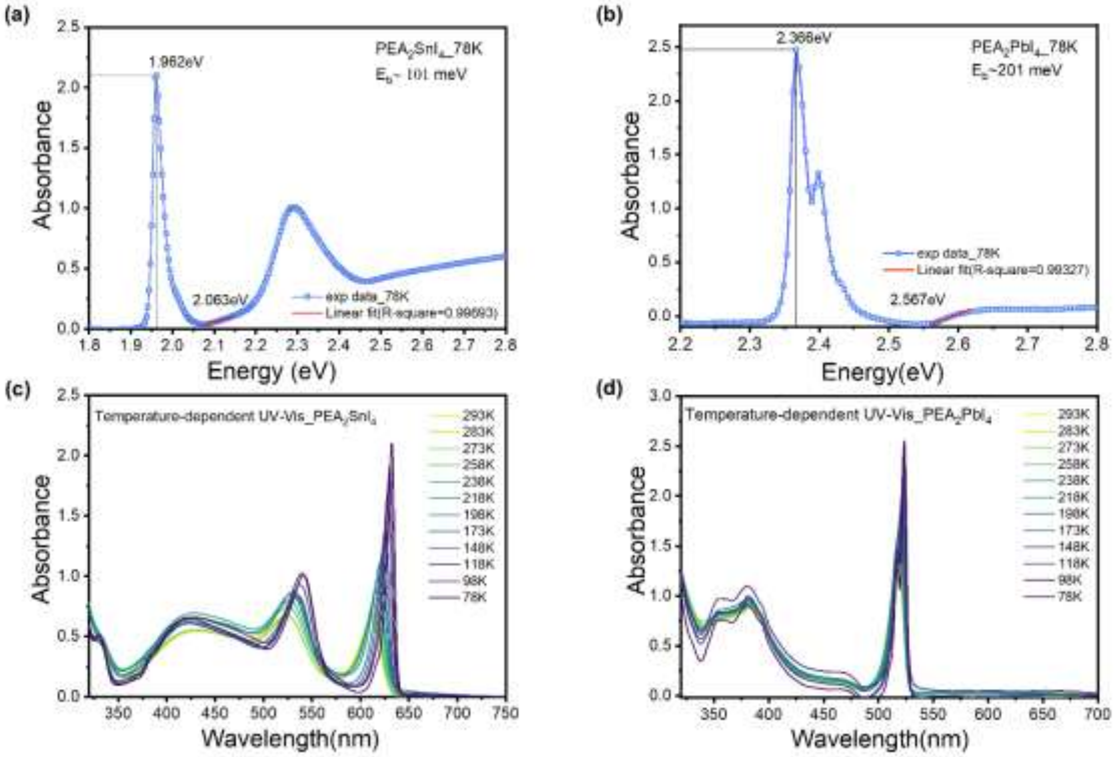

Figure S16 (a)(b). The absorption spectrum of PEA_2SnI_4 at low temperature exhibits an excitonic resonance at 1.962 eV, well separated from the continuum edge at 2.063 eV, indicating an exciton binding energy of $E_b \approx 101$ meV, like what is observed in its PEA_2PbI_4 ($E_b \approx 201$ meV). (c)(d). Temperature-dependent UV-vis absorption spectra in PEA_2SnI_4 and PEA_2PbI_4 thin films both show that the excitonic resonance is well separated from the continuum edge at different temperatures.

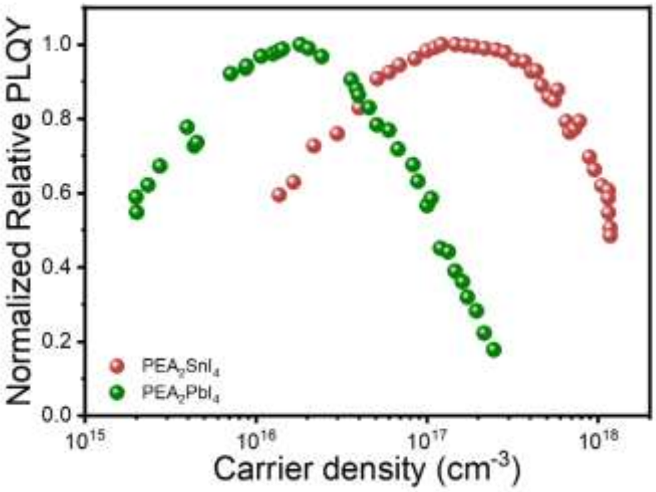

Figure S17. Relative photoluminescence quantum yield (PLQY) of PEA_2SnI_4 and PEA_2PbI_4 at room temperature.

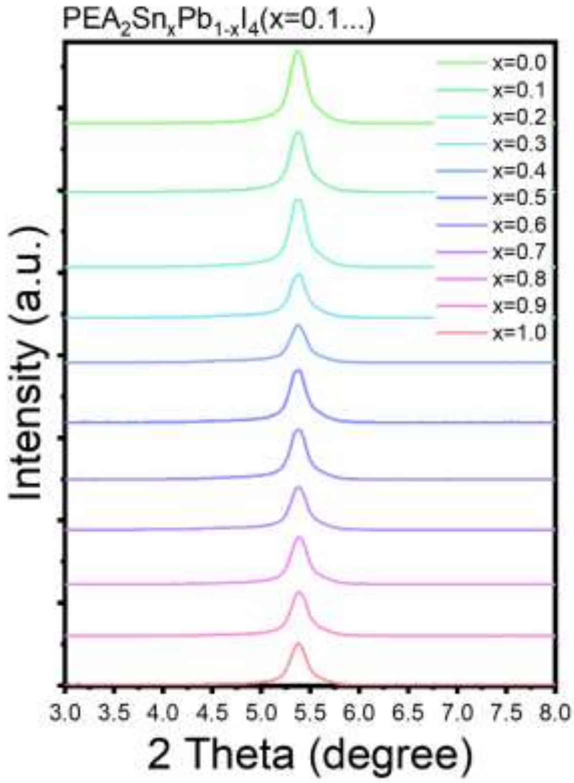

Figure S18. X-ray diffraction (XRD) of $PEA_2Sn_xPb_{1-x}I_4$(x=0.1...) polycrystalline films.

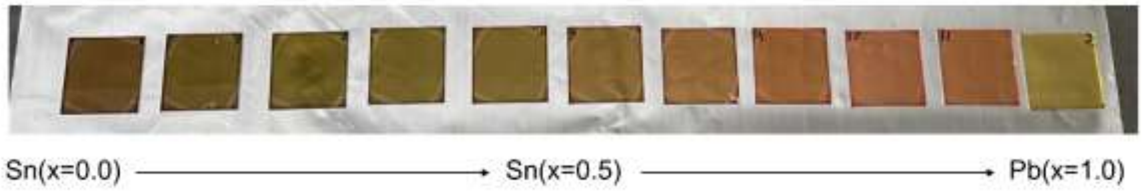

Figure S19. The picture of samples in $PEA_2Sn_xPb_{1-x}I_4$ (x=0.1...) polycrystalline films.

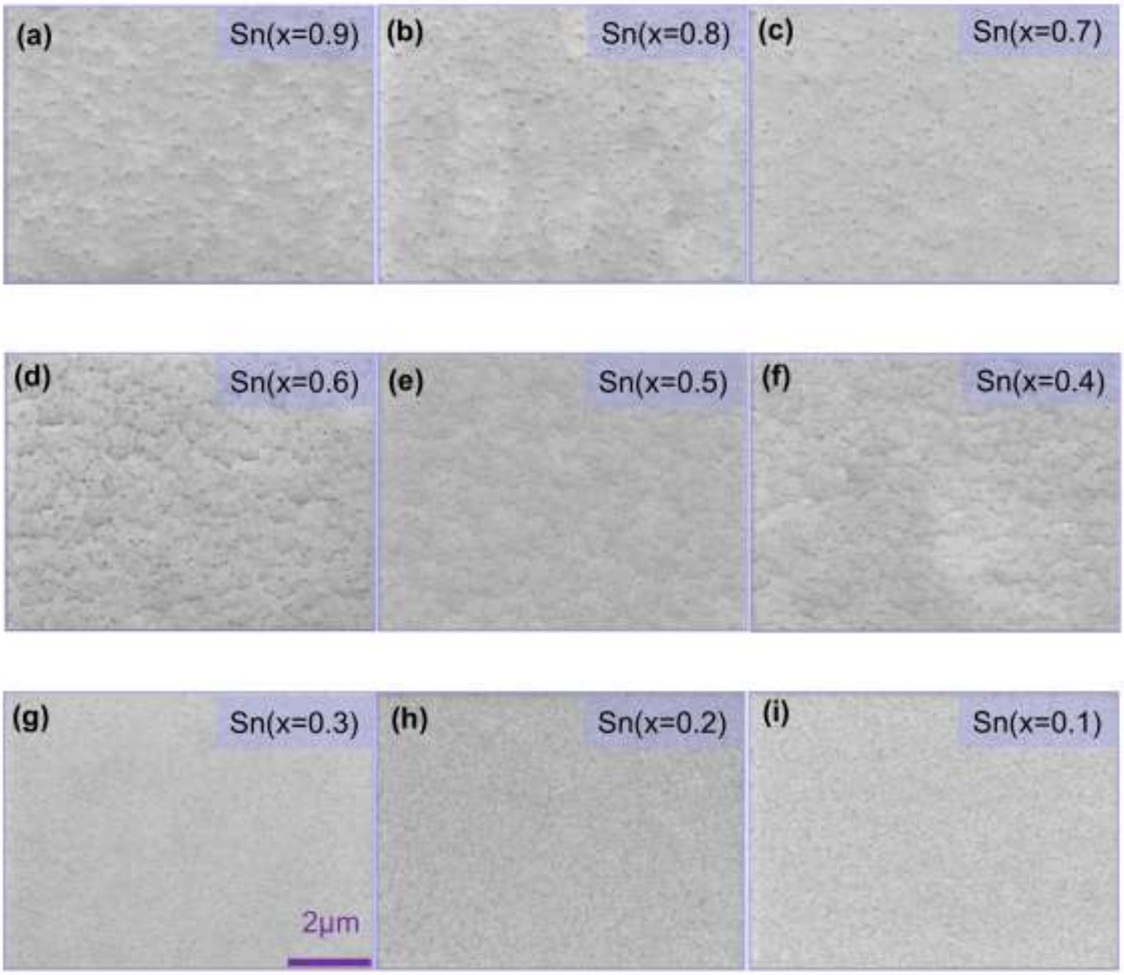

Figure S20 (a-i). Scanning electron microscope (SEM) images with the same magnification (30 kx) of $PEA_2Sn_xPb_{1-x}I_4$ (x=0.1…) polycrystalline films show that the grain size becomes smaller with the Pb ratio increasing.

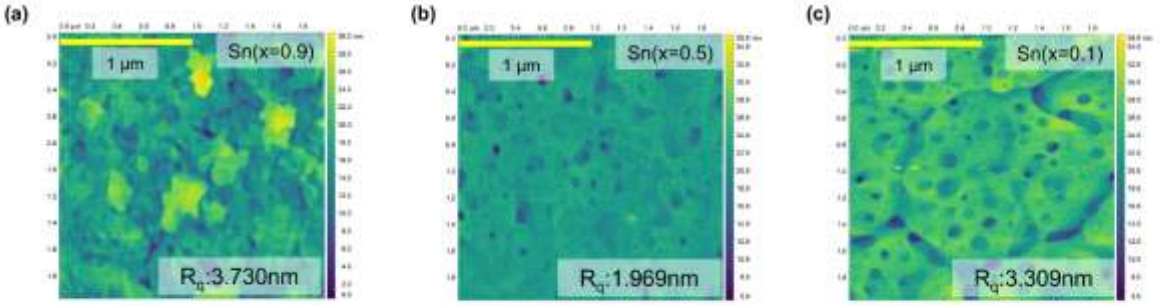

Figure S21 (a-c). Atomic force microscopy (AFM) images (2 μm×2 μm) of Sn(x=0.9), Sn(x=0.5), and Sn(x=0.1), which have come from the series $PEA_2Sn_xPb_{1-x}I_4$(x=0.1…), show more pin holes with the Pb ratio increasing.

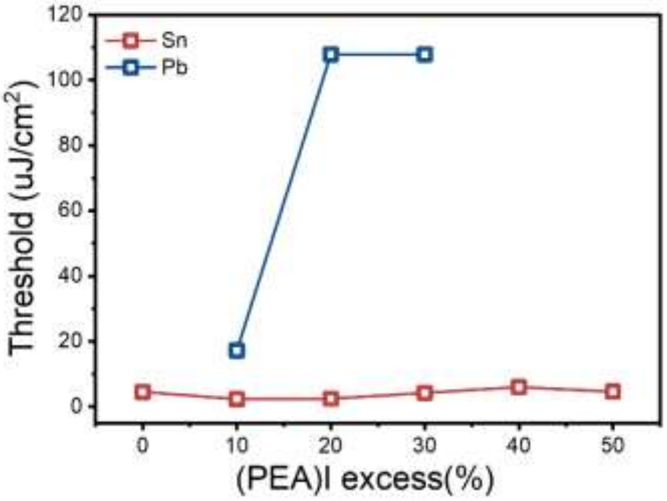

Figure S22. ASE threshold of PEA_2SnI_4 and PEA_2PbI_4 as a function of the (PEA)I excess used in the precursor solution. The (PEA)I excess is reported in percentage respect to the stoichiometric amount.

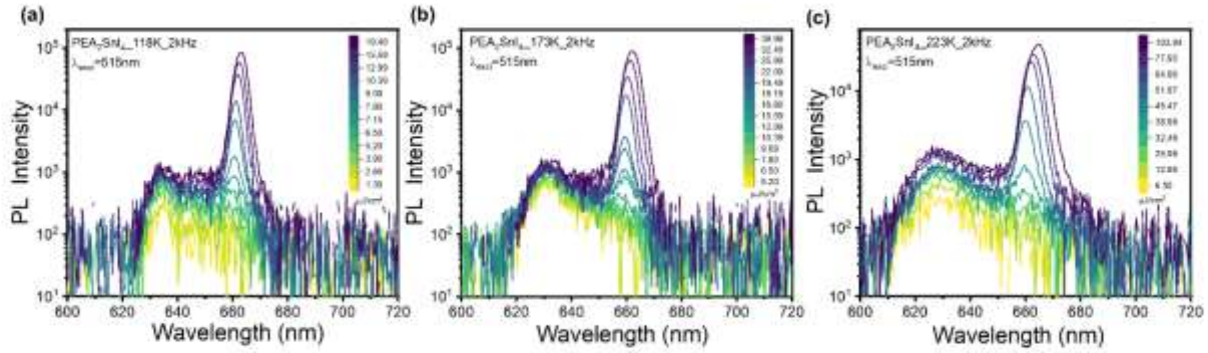

Figure S23 (a). Under the same pump condition (λ_{exci}=515 nm, repetition rate 2 kHz, pulse duration 300 fs), pump fluence-dependent PL Spectra of PEA_2SnI_4 at 118 K with the pump fluence increases from 1.3 to 19 μJ/cm^2. (b).at 173 K with the pump fluence increases from 5 to 39 μJ/cm^2. (c). and at 223 K with the pump fluence increases from 7 to 105 μJ/cm^2.

Table S2. Bi-exponential decay parameters, and fast and slow delay of TRPL analysis of Sn and Pb at 293 K and 78 K.

Temperature (K)	Samples	A1	π1（ps）	A2	π2（ps）
293	PEA_2SnI_4	0.46	30.71	0.44	490.47
	PEA_2PbI_4	0.63	65.63	0.37	165.28
78	PEA_2SnI_4	0.92	47.27	0.08	174.31
	PEA_2PbI_4	0.53	402.19	0.47	510.56

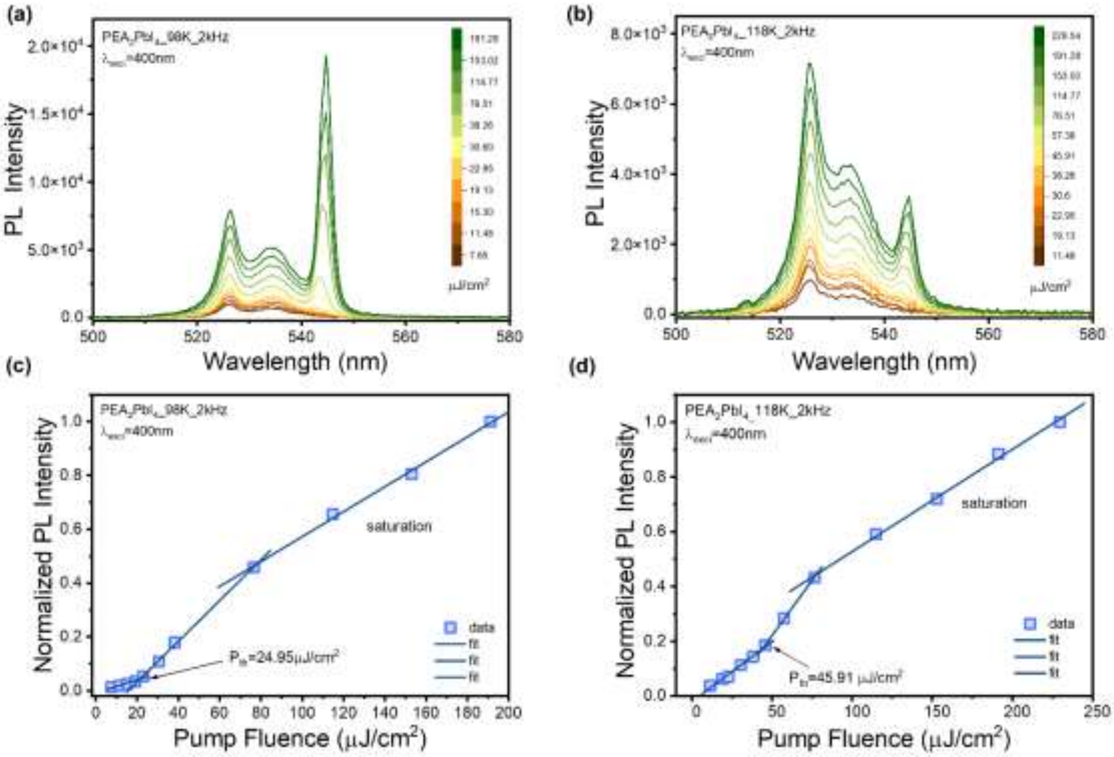

Figure S24 (a). Under the same pump condition (λ_{exci}=400 nm, repetition rate 2 kHz, pulse duration 300 fs), pump fluence-dependent PL Spectra of PEA_2PbI_4 at 98 K with the pump fluence increases from 8 to 191 μJ/cm^2. (b) at 118 K with the pump fluence increases from 11 to 230 μJ/cm^2. (c). The PL intensity as a function of pump fluence of Pb at 98 K, which observed the ASE threshold is 25 μJ/cm^2, and the linear growth is no longer observed due to ASE saturation at higher pump fluence. (d). The PL intensity as a function of pump fluence of Pb at 118 K shows the ASE threshold up to 46 μJ/cm^2, and the linear growth is no longer observed due to ASE saturation at higher pump fluence.

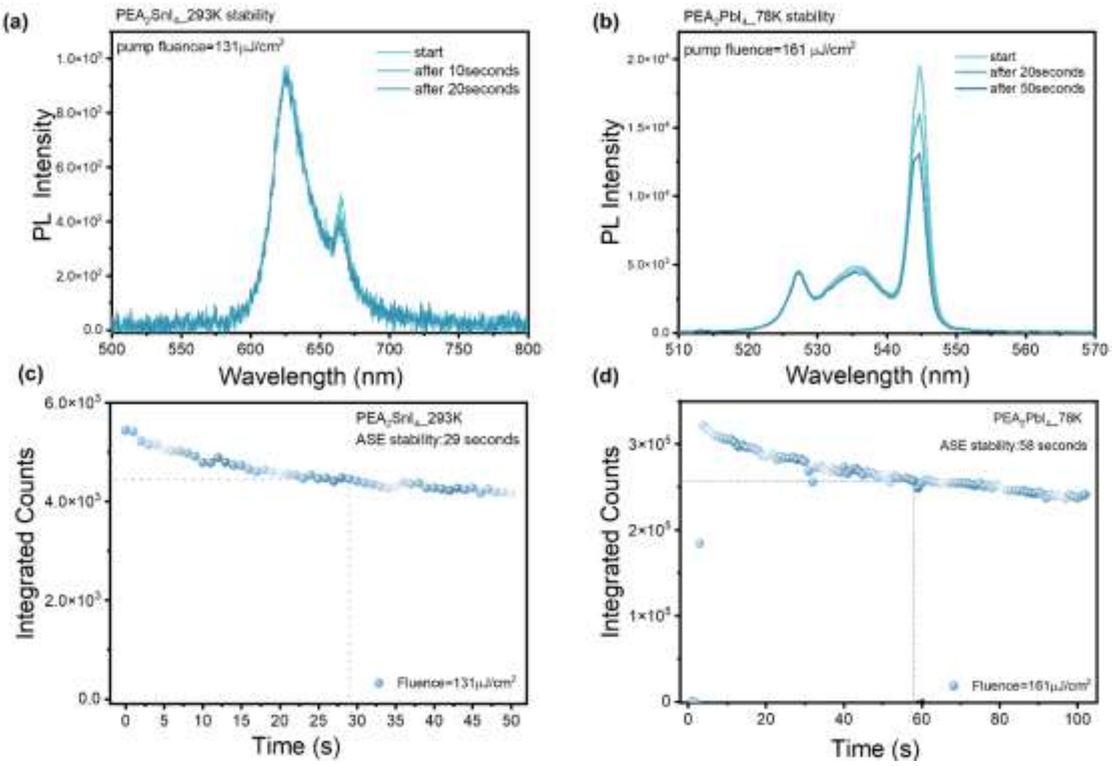

Figure S25 (a)(c). The ASE PL spectra at the different exposure times, and the ASE stability of PEA_2SnI_4 under continuous exposure to a 515 nm excitation laser (pulse width 300 fs, repetition rate 2 kHz) at 131 μJ/cm^2 at 293 K. (b)(d). The ASE PL spectra at the different exposure times, and the ASE stability of PEA_2PbI_4 under continuous exposure to a 400 nm excitation laser (pulse width 300 fs, repetition rate 2 kHz) at 161 μJ/cm^2 at 78 K.

References:

(1) Zhang, T.; Zhou, C.; Feng, X.; Dong, N.; Chen, H.; Chen, X.; Zhang, L.; Lin, J.; Wang, J. Regulation of the Luminescence Mechanism of Two-Dimensional Tin Halide Perovskites. *Nat Commun* **2022**, *13* (1), 60. https://doi.org/10.1038/s41467-021-27663-0.

(2) Li, Y.; Zhou, H.; Gong, Z.; Xia, M.; Han, Y.; Sheng, X.; Wang, T.; Wang, H.; Zhu, H.; Shi, E. Photo-Excited Carrier Behaviors of Two-Dimensional Tin Halide Perovskite Single Crystals. *Cell Rep. Phys. Sci.* **2024**, *5* (6). https://doi.org/10.1016/j.xcrp.2024.102020

(3) Bloembergen, N.; Purcell, E. M.; Pound, R. V. Relaxation Effects in Nuclear Magnetic Resonance Absorption. Phys. Rev. 1948, 73 (7), 679–712. https://doi.org/10.1103/PhysRev.73.679.

(4) Fabini, D. H.; Siaw, T. A.; Stoumpos, C. C.; Laurita, G.; Olds, D.; Page, K.; Hu, J. G.; Kanatzidis, M. G.; Han, S.; Seshadri, R. Universal Dynamics of Molecular Reorientation in Hybrid Lead Iodide Perovskites. J. Am. Chem. Soc. 2017, 139 (46), 16875–16884. https://doi.org/10.1021/jacs.7b09536.
